# Male- and Female-Biased Gene Expression of Olfactory-Related Genes in the Antennae of Asian Corn Borer, *Ostrinia furnacalis* (Guenée) (Lepidoptera: Crambidae)

**DOI:** 10.1371/journal.pone.0128550

**Published:** 2015-06-10

**Authors:** Tiantao Zhang, Brad S. Coates, Xing Ge, Shuxiong Bai, Kanglai He, Zhenying Wang

**Affiliations:** 1 State Key Laboratory for Biology of Plant Diseases and Insect Pests, Institute of Plant Protection, Chinese Academy of Agricultural Sciences, Beijing 100193, China; 2 United States Department of Agriculture, Agricultural Research Service, Corn Insects & Crop Genetics Research Unit, Iowa State University, Ames, IA, 50011, United States of America; Ghent University, BELGIUM

## Abstract

The Asian corn borer (ACB), *Ostrinia furnacalis* (Guenée), is a destructive pest insect of cultivated corn crops, for which antennal-expressed receptors are important to detect olfactory cues for mate attraction and oviposition. Few olfactory related genes were reported in ACB, so we sequenced and characterized the transcriptome of male and female *O*. *furnacalis antennae*. Non-normalized male and female *O*. *furnacalis* antennal cDNA libraries were sequenced on the Illumina HiSeq 2000 and assembled into a reference transcriptome. Functional gene annotations identified putative olfactory-related genes; 56 odorant receptors (ORs), 23 odorant binding proteins (OBPs), and 10 CSPs. RNA-seq estimates of gene expression respectively showed up- and down-regulation of 79 and 30 genes in female compared to male antennae, which included up-regulation of 8 ORs and 1 PBP gene in male antennae as well as 3 ORs in female antennae. Quantitative real-time RT-PCR analyses validated strong male antennal-biased expression of *OfurOR3*, *4*, *6*, *7*, *8*, *11*, *12*, *13* and *14* transcripts, whereas *OfurOR17* and *18* were specially expressed in female antennae. Sex-biases gene expression described here provides important insight in gene functionalization, and provides candidate genes putatively involved in environmental perception, host plant attraction, and mate recognition.

## Introduction

The olfactory and chemosensory systems of Lepidoptera are important for several biologically-important functions including adult mate attraction, oviposition site selection and host plant preference, and negative taxis [1]. Trichoid sensilla are located on moth antennae and composed of pore tubes through which volatile hydrophobic odorants from the environment can enter. The specific detection and subsequent behavioral responses to environmental volatiles are mediated by the initial binding and transport of hydrocarbons across the aqueous sensillar lymph by classes of odorant binding proteins (OBPs) and chemosensory proteins (CSPs). The OBPs are small hydrophilic proteins that have a conserved tertiary protein structure of 6 alpha-helices coordinated by 3 disulfide bridges [2], and CSPs have 4 alpha-helices that form 2 disulfide bridges [3]. Both OBPs and CSPs are localized in the sensillar lymph of trichoid sensilla [4]. CSP sequences are comparatively more highly conserved, whereas OBPs have diverged and are classified into subfamilies based on functional and phylogenetic evidence: Classic, Dimer, D7, pheromone binding proteins (PBPs)/general odorant binding proteins (GOBPs), chemical-sense-related lipophilic-ligand-binding proteins (CRLBPs), antennal binding protein group I (ABPI) and II (ABPII), and atypical Plus-C and Minus-C OBPs [5,6]. OBPs can retain little intraspecific homology outside of six conserved cysteine residues, but those that are missing the C2 cysteine are referred to as the minus-C OBP class and those with an excess of cysteines belong to the plus-C OBP class. Both OBPs and CSPs perform analogous functions of bind chemical cues encountered in the environment and transporting these cues within chemosensory tissues to receptors located at the neuron surface.

The ligands that are bound by a majority of OBPs, including the lepidopteran PBP subfamily, remain largely unknown nor are the subsequent behavioral responses fully understood, with the possible exception of GOBPs and other OBPs which may function in host plant volatile recognition, taste [7] or xenobiotic perception [8]. The first described OBP was an *Antheraea polyphemus* PBP that bound radioactively labeled sex pheromones from conspecific females and was hypothesized to function in the perception of conspecific pheromones [4]. Indeed, PBPs were shown to bind sex pheromones *in vitro* and subsequently hypothesized to play a potential role in the discrimination of pheromone cues by male Lepidoptera [9,10]. These PBPs may be involved in the pH-dependent binding and transport of female sex pheromones to odorant receptors (ORs) on olfactory receptor neurons (ORNs). Specifically a pH-dependent conformational change was shown to shift the position of the C-terminal tail in or out of the hydrophobic PBP binding pocket from *Bombyx mori* [11], *Amyelois transitella* [12], and *Antheraea polyphemus* for PBPs [13], which may be a feature common of lepidopteran PBPs [14]. At neutral pH pheromones are predicted to bind a PBP hydrophobic binding pocket, which allows diffusion of hydrocarbons across the sensillar lymph and prevention of breakdown by pheromone-degrading enzymes [14]. Similarly, Große-Wilde et al. suggested that PBPs of *B*. *mori* can mediate the bombykol-induced activation of BmOR1 [10], and subsequently showed that OBPs promote pheromone sensitivity in a ligand-specific manner [15]. Directional selection between orthologs of male *Ostrinia nubilalis* and *O*. *furnacalis* antennal expressed PBP3 was hypothesized to result from the evolution of selective binding between structurally distinct sex pheromones emitted by corresponding females of the same species [16]. Furthermore, neuron response to the *Drosophila* pheromone, *cis*-vaccenyl acetate (cVA), was shown to directly depend on the function of an OBP called LUSH [17,18] that acts to solubilize and transport cVA [19]. Analogous OBP polymorphisms have also been shown to elicit variant behavioral responses in *Drospohila* [20,21]. Recent studies have reported electrophysiological response to the odorant indole decrease when the *Anopheles gambiae* AgamOBP1 was knocked down using RNAi [22]. However, it remains unclear how broadly these dependencies apply to other insect systems since seemingly contradictory studies have reported that silk moths can respond to the pheromone bombykol in absence of the cognate PBP [23,24].

Insect OBPs and CSPs perform analogous roles in that both reversibly binding small ligands with dissociation constants in the micromolar range, despite differences in structure [25] and biological function. Similar to the PBP subfamily of OBPs, CSP conformational changes are predicted when in association with cognate ligands [26]. Thus is has been hypothesized that CSPs may be involved in insect chemical communication, although most specific functions have not yet been discovered. CSPs are expressed in a variety of tissues and may be have evolved a divergent cellular functions involved in environmental perception [27]. For example, CSPs expressed in the chemosensory sensilla [27] are believed to detect environmental carbon dioxide levels [28] and modulate behavioral phase changes in the migratory locust [29]. The transduction of chemical cues from the environment to neurons likely involves a pathway analogous to OBPs, but the function of this portion of the insect chemosensory system also remains largely unknown.

Antennal-mediated olfactory detection in Lepidoptera involves ORNs that project into the sensillar lymph of trichoid sensilla, where specifically-tuning of each neurons is achieved by the expression of a specific OR. Each OR forms a voltage-gated ion channel following hetero-dimerization with OR2, which is also referred to as the odorant receptor co-receptor (*Orco*) and is an ortholog of the *Drosophila melanogaster* OR, DmOr83b. Species in the genus *Ostrinia* are a model for the study of the olfactory system of Lepidoptera, and has been used investigate the selectivity and response of ORs to sex pheromones in order to understand the general mechanisms of chemosensory response. Female *O*. *nubilalis* and *O*. *furnacalis* respectively produce and emit a blend of E/Z-11-tetradecenyl acetate (E/Z11-14:OAc) and E/Z-12-tetradecenyl acetate (E/Z12-14:OAc), which evoke responses by males of the corresponding species. The ORNs of male *Ostrinia* are classified with respect to strength of impulses produced in laboratory electophysiological recordings when stimulated by female pheromones; large, medium, and small spiking neurons. Large spiking ORNs in *O*. *furnacalis* respond to both E- and Z-12-14:OAc components of conspecific female pheromone blends, whereas medium spiking neurons responded with equal intensities to Z9-, E11- and Z11-14:OAc. Analogously, *O*. *nubilalis* large spiking ORNs specifically responded to intraspecific female pheromone components [30].

The molecular basis of these specific ORN responses have been partially elucidated in relation to species-specific male behavioral responses. Pheromone stimulation of co-expressed *Orco* and other OR proteins (ORx) in the *Xenopus* oocyte system are used to assay for specific responses by measuring changes in membrane ion permeability. These two-electrode voltage clamp electrophysiology measures indicate that *O*. *nubilalis* OR6 and OR2 (*OnOR*6*/2*) respond specifically to Z11-14:OAc, but *OnOR3/2* and *5/2* respond to the known antagonist Z9-14:OAc as well and female *O*. *nubilalis* E11-, Z11-14:OAc and *O*. *furnacalis* emitted E12- and Z12-14:OAc [31]. Due to independent isolation, it should be noted that the nomenclature of *Ostrinia* ORs has the *Ofur*OR4 described by Miura et al. [32] being the direct ortholog of *OnOR3* and *OfurOR3* from other studies [33]. More importantly, *Xenopus* oocyte assays showed that *OnOR3* and *OfurOR3* in complex with *Orco* produce species-specific electrophysiological responses when stimulated by corresponding female sex pheromones, and thus hypothesized to be the ORs expressed in large spiking ORNs [33]. Site-directed mutagenesis of amino acids at position 148 of *OfurOR3* from an alanine to the serine in *OnOR3* resulted in the electrophysiological response of the mutant *OfurOR3* to *O*. *nubilalis* female pheromones, and linked the specific change to functional variation in species-specific pheromone responses [33]. The aforementioned proteins may interact with sensory neuron membrane proteins (SNMPs) and ionotropic receptors (IRs) for olfactory signal transduction, and odorant degrading enzymes (ODEs) [1,34] to restore the sensitivity of the sensory neuron [35].

Despite these advances in the elucidation of sex pheromone perception and ORN response, little is known regarding the specific function(s) of many OBPs, their cognate ORs, nor the behavioral responses these sensory pathways elicit. OBPs have been characterized from fully assembled genomes, and wherein they comprise a diverse gene family with complex evolutionary histories that likely may have resulted from a high degree of functional diversification [36]. For example, 44 OBPs are encoded by 6 tandem duplicated gene clusters in the *B*. *mori* genome indicating that paralogs may have evolved diverged functions involved in chemosensory detection [37], and have undergone an enigmatic path of gene gain and loss compared to other arthropods. Differential expression of genes involved in the lepidopteran olfactory system may be important for understanding the evolution of this duplicated gene family, and well as potentially uncovering the molecular basis for variation in moth response to potential mates, host plants and selection of oviposition sites. Sex biased expression of gene family members is an example of subfuncationalization [38], wherein duplicated genes may be retained in the genome due to the derivation of novel expression patterns and reinforced by exclusionary sex-specific functions. The maintenance of sex-biased gene expression may be under strong positive selection in instances where a gene function in sexual attraction and mating, where loss of this fidelity may negatively impact reproduction. For example, male antennal-specific *Manduca sexta MsexOR-1* and *MsexOR-4* are suggested to function in ORN response to sex pheromone [39]. Of the 48 OR genes identified in the *B*. *mori* genome by Wanner et al. [40], *BmOR3* was expressed 6 to 8-times higher in females and 12 OR transcripts expressed predominantly in female antennae, and only 3 ORs were shown to be male antennae-specific [40]. Analogous genomic studies are yet to report system-wide sex-biased gene expression analyses in Lepidoptera, neither have comparative orthologies to *B*. *mori* olfactory genes been previously identified in another moth species.

Expressed sequence tag (EST)/transcriptome approaches have also been used to identify chemosensory receptors in arthropod species and present an alternative approach when full genome sequence assemblies are unavailable [41–45], and have proven to be valuable for elucidating olfactory system function. The motivation for this study was to use a transcriptomic approach to identify gene components of the *Ostrinia* antennal olfactory system (CSPs, OBPs and ORs), and apply sex-biased expression data to formulate hypotheses for future functional genomic research. Our prediction of orthologous gene relationships between *O*. *furnacalis* and *B*. *mori* provide a valuable tool for comparative genomic and function analyses. This study provides important tissue- and sex-biased gene expression data of olfactory-related genes in the antennae of *O*. *furnacalis* and define putative one-to-one orthologous gene relationships in Lepidoptera for comparative functional analyses. Results of this study are discussed in a system-wide evolutionary context wherein expression bias has precluded or reinforced the functional diversification of lepidopteran olfactory response pathways, and gene regulatory changes in multiple system components may act in concert to modulate sex-specific behaviors.

## Materials and Methods

### Insects rearing and antennae collection

Pupal *O*. *furnacalis* were obtained from a laboratory colony at the Institute of Plant Protection, Chinese Academy of Agricultural Sciences, Beijing, China. Male and female pupae were placed into different gauze cages for eclosion respectively. After emergence adults were fed with cotton dipped in a 10% honey solution, and the solution was renewed daily. Antennae from 3-day old adults of both sexes were sampled separately, pooled by sex and flash frozen in liquid nitrogen. Antennae, maxillary palpus and legs of both sexes were also collected and put into liquid nitrogen for downstream real-time quantitative RT-PCR (RT-qPCR) validation of gene expression.

### Antennal transcriptome assembly and functional gene annotation

Total RNA from male and female antennae was extracted separately using TRIzol reagent (Invitrogen) according to manufacturer instructions. Subsequent cDNA library construction and Illumina sequencing was performed at SinoGenoMax Co., Ltd, Beijing, China. Briefly, enrichment of mRNA from ~20 μg of total RNA used oligo (dT)25 magnetic beads, and 1^st^ strand synthesis was conducted using AMV reverse transcriptase primed with an oligo (dT) primer followed by 2^nd^-strand cDNA synthesis by using random hexamer-priming for reactions including RNase H and DNA polymerase I. The cDNA was fragmented, end-repaired, and ligated with library-specific barcoded adaptors. Library fragments were PCR amplified a minimum number of cycles in order to avoid normalization for downstream quantitative gene expression analyses. Amplified products were purified with QIAGEN MiniElute PCR Purification Kit (Qiagen, Venlo, Netherlands), and approximately equal molar proportions of male and female indexed libraries were sequenced on a single flow cell of an Illumina HiSeq 2000. Each library was sequenced in a second technical replicate on an independent Illumina HiSeq 2000 lane with an approximate equal molar ratio of each library loaded. Raw sequence data from all runs were obtained in fastq format.

### Assembly and functional gene annotation

Illumina output (fastq formatted read data) were trimmed for quality scores (*q*) < 20. A single *de novo* assembly of the combined reads was performed from trimmed read data using the short read assembler ABySS [46]. To assess assembler performance, assemblies of all the reads were compared for *k* values ranging from 26 to 50 bp [47]. CD-HIT-EST was used to clusters similar sequences and removed redundant segments using the web interface at http://weizhong-lab.ucsd.edu/cdhit_suite/cgi-bin/index.cgi?cmd=cd-hit-est [48], and the longest contig sequences were retained as the best assembly result. The program Getorf from the EMBOSS package [49] was used to predict and extract the longest open reading frames (ORFs) in assembled contig sequences. Since splice variants and sequence heterogeneity in UTRs tended to uncouple contigs belonging to the same gene, an all-versus-all tblastn search was performed (*E*-value cutoff ≤ 10^−50^, and protein identities ≥ 95%), and putatively homologous sequence data were aligned using the CLUSTAL W algorithm and inspected manually.

Functional gene annotations were collected for all contig (unigene) sequences ≥ 150 bp using Blast2GO [50], where initial searches of the National Center for Biotechnology Information (NCBI) non-redundant (nr) protein database were conducted with the BLASTx algorithm, followed by collection of gene ontology (GO) terms from the GO database and retrieval of KEGG Pathway designations. The BLASTx output was then processed with wapRNA [51]. Contigs receiving putative annotation as OR gene family members were investigated in greater detail. Assembly of contigs from iterative ABySS assemblies were achieved using CAP3 (default parameters; [52]), and were re-annotated by manual blastx of the NCBI protein database and search of conserved domain database (CDD) and protein family (pfam) databases (http://pfam.xfam.org/search).

### Analysis of differential gene expression

Differential gene expression and transcript bias between male and female antennal read data were conducted by independent alignment of short reads from male- and female-specific libraries to the reference antennal transcriptome assembly. Specifically, the Burrows-Wheeler Alignment (BWA [53]) was used to align reads to the reference antennal transcriptome (≤ 5 allowed mismatches), and expression level for each gene was initially calculated as Reads Per Kilobase per Million mapped reads (RPKM) using an in-house Perl module [51]. The RPKM method eliminates the influence of different gene lengths and sequencing discrepancies when calculations of expression abundance are made [54], and corrected variance in RPKM values between male and female alignments were normalized based on the depth of reads aligned to the housekeeping gene beta-actin. Since RPKM estimates of differentially expressed genes (DEGs) show bias towards overestimation of read count and transcript length, a different method was used to detect significant variation between *O*. *furnacalis* male and female antennal transcript levels. The algorithm DESeq assumes most transcripts do not represent DEGs, and implements a scaling factor which is calculated as the median ratio of read counts for each gene as a ratio of the geometric mean across all replicated libraries. The DESeq Bioconductor package v.1.6.0 for the R statistical package [55–57] was used here to construct the MA-plot-based method with random sampling model to identify DEGs. Differences in transcript abundances between male- and female-specific libraries were plotted on a log_2_(fold-change) scale and significance thresholds set at > 5-fold [50].

The level of transcripts *OfurOR1* to *20* were estimated in male and female antennae, maxillary palpus and legs using real-time RT-qPCR, and the subset inclusive of the pheromone receptor subfamily used to validate RNA-seq estimates of gene expression. Total RNA for each tissue was prepared as described above, and then treated with DNase I (Invitrogen, New York, USA) to remove trace amounts of genomic DNA prior to cDNA synthesis. First strand cDNAs were synthesized by use of the AMV Reverse Transcription Kit (Promega, Wisconsin, USA). Primers for the *O*. *furnacalis* β-actin gene (accession number: GU301782) and 20 putative annotated OR genes were designed using Primer Express (ABI, USA), and respectively used to amplify target genes and a reference gene in separate reactions (**S1 Table**). Quantitative RT-qPCR was performed using an ABI Stepone Plus instrument with 20.0 μl reaction mixtures containing 10.0 μl SYBR Green qPCR Master Mix, 1.0 μl each of primers (10μM), 1.0 μl first strand antennal, maxillary palp or leg cDNA template and 7.0 μl ddH_2_O. Reactions were setup in triplicate for each template across all primer pairs (technical replicates), and repeated for 3 independent samples (biological replicates). The PCR program was as follows: 95°C for 2 m, followed by 40 cycles of 95°C 10 s and 60°C for 40 s, and then melt curve analysis was performed to test locus-specificity of reaction products. The data were analyzed by the comparative 2^-∆∆CT^ method [58], with transcript levels normalized by comparison C_T_ estimates from the beta-actin reaction. Transcript expression levels between male and female were compared as fold-change using the male antennae levels arbitrarily set at one, and significance of any relative difference between male and female expression (two conditions) was determined using paired T-tests as described by Pabiner et al., [59]. The correlation analysis was executed by using cor function of the R Statistical Package, and regression analysis was executed with the lm function [60].

### Phylogenetic analysis of olfactory-related proteins

The *O*. *furnacalis* contigs that received functional annotations as putative OR-, OBP-, and CSP-like genes were retrieved from our reference antennal transcriptome assembly, and derived *O*. *furnacalis* amino acid sequence and putative translations for transcripts were made using Getorf from the EMBOSS package [49]. OR protein sequences previously identified in lepidopteran insects were downloaded from GenBank (71 from *Bombyx mori* and 1 from *Conogethes punctiferalis*, respectively). Nomenclature for all *B*. *mori* OR orthologs were retained from previously published analyses; *Bmor*OR1-48 [40] *BmorOR19j*, *22j*, *23j* [61]; *BmorOR49*-*68* [62]). A multiple amino acid sequence alignment was generated from downloaded and *O*. *furnacalis* ORs using the MUSCLE algorithm implemented using default parameters of MEGA 5.2.2 [63]. Alignments were similarly constructed for OBP using 10, 5, 19, 17, 11 sequences respectively from GenBank accessions for *Chilo suppressalis*, *Manduca sexta*, *Helicoverpa armigera*, *Spodoptera exigua and B*. *mori*. The nomenclature among *B*. *mori* OBP orthologs was used as described by Gong et al. [37]. The find best model of sequence evolution option of MEGA 5.2.2 [40] was used to evaluate both aligned OR and CSP sequences.

Phylogenetic reconstructions for OR and CSP orthologs were performed independently using neighbor-Joining (NJ) methods to evaluate the amino acid sequence evolution using the Jones-Taylor-Thorton (JTT) model, with node support generated from 1,000 bootstrap pseudoreplcations of the data. *OfurOR13*, *40*, *45*, *46*, *47*, *50*, *53*, *54*, and *55* were omitted due to short derived protein sequence. Among site rate variation was accounted for with a gamma distribution of 4.994 and 3.599 respectively for the OR and CSP trees. Lepidopteran CSP protein family phylogenies used the neighbor-joining (NJ) method with Poisson correction of genetic distances. Trees were not rooted. All phylogenetic analyses used MEGA 5.2.2 [63].

## Results

### RNA preparation, cDNA library construction and Illumina sequencing

High quality total RNA preparations were obtained from both adult *O*. *furnacalis* male and female antennal samples and cDNAs were successfully synthesized from mRNA enriched fractions. A total of 7, 990, 984, 106 bp and 5, 286, 885, 848 bp of sequence data were respectively obtained from male and female antennal specific cDNAs libraries on an Illumina HiSeq 2000. Raw reads from both Illumina HiSeq 2000 runs for the female and male antennal libraries were submitted to the GenBank Short Read Archive (SRA) under respectively accession numbers SSR1222986 and SRR1226611.

### Antennal transcriptome assembly and functional gene annotation

A combined assembly of these two datasets resulted in 37,687 contigs of ≥ 300 bp, which had with a mean length of 818 bp and the N50 length of 1,022 bp (3.02 million bp). A total of 37,687 clusters were obtained from CD-HIT (**S1 Text**), and represented the longest ORFs for each transcript. Among the 37,687 assembled *O*. *furnacalis* transcripts, a total of 15,544 showed “hits” following BLASTx homology search to the NCBI non-redundant (nr) protein database (*E*-value cut-off ≤ 10^−5^; **S2 Table**). These BLASTx search results identified a total of 89 transcript sequences with putative homology to olfactory-related genes; 56 ORs (**Table 1**) and 23 OBPs (Table A in **S3 Table**), and 10 CSPs (Table B in **S3 Table**). Of these transcripts, 14 showed > 95% sequence identity to previously identified *O*. *furnacalis* gene products already represented in GenBank: 5 PBPs (Accession number: GU828024 to GU828028; 1 GOBP2 (DQ673101) and 8 ORs (AB467327, JX910526, JN169134, JN169136, JN169138, JN169142, JX910532, JX910533; [16,32]; remaining search results not shown). The additional 75 putative olfactory-related transcripts found in the current study were not previously described in *O*. *furnacalis* or other species of *Ostrinia*. Of the 56 *OfurOR* transcripts (**File A in S2 Text**), the putative complete CDS was obtained for 13 (**File B in S2 Text**), wherein C-terminal CDS was obtained with greater prevalence. GO annotations from the longest sequence in each CD-HIT cluster ("UniGenes") were used to obtain function gene annotations. These annotations showed a high percentage of transcripts in GO Level 1 Cellular Component, GO Level 2; cell part (86.78%), cell (86.78%), and organelle (80.17%). Additionally, GO Level 1 Molecular Function showed the highest proportion of annotations in GO Level 2 binding (64.86%), and GO Level 1 Biological Process showed the greatest number of annotations in cellular process (76.03%) and metabolic process (76.03%). Contigs that received putative OR gene family member annotation were investigated in greater detail, where CDD and pfam database search results indicated that all 56 putative *OfurOR*s encode 7tm_6 motifs which comprise 7 transmembrane domains that are typical of receptor proteins (pfam02949; superfamily cl20237; remaining data not shown).

**Table 1:** *Ostrinia furnacalis* assembled Unigenes with annotation as candidate olfactory receptors (*OfurOR*s).

					M:F	M:F
Gene	Contigs	Residue	Top blastx hit	% ID	RSq	qRT
*OfurOR1*		424	BAH57982.1| *Ostrinia furnacalis* OR1	100	NPF	3.04
*OfurOR2*/*ORco*	k58_921629 k80_505084	473	AGG91643.1|olfactory receptor OR2	99	1.35	4.42
	k74_603809 k80_492746					
*OfurOR3*	k66_754859 k42_1382723	422	AFK30395.1| *Ostrinia furnacalis* OR3	100	35.87	35.61
*OfurOR4*	k68_715603	425	AFK30397.1| *Ostrinia furnacalis* OR4	100	2.45	27.81
*OfurOR5a*	k58_921629	227	AGG91646.1| *Ostrinia furnacalis* OR5a	100	18.06	28.3
*OfurOR5b*	k60_883275	288	BAI66613.1| *Ostrinia furnacalis* OR5a	100		
*OfurOR6*	k56_956826	421	AFK30403.1| *Ostrinia furnacalis* OR6	100	29.08	74.33
*OfurOR7*	k46_1228889 k68_706960	428	AGG91649.1| *Ostrinia furnacalis* OR7	99	6.86	54.88
*OfurOR8*	k76_574193, k80_503264	408	BAI66616.1| *Ostrinia furnacalis* OR8	100	34.29	34.88
	k80_497670					
*OfurOR9*	k38_311779 k68_581233 k76_564123	448	AB186511.1 BmOR9	67	0.52	18.97
*OfurOR10*	k40_1435593	271	BAH66346.1 | BmOR41	51	0.74	7.72
*OfurOR11*	k78_351090 k60_548503	170	XM_004929519.1|BmOR85f	46	0.3	31.16
*OfurOR12*	k38_720713	246	AB472104.1|BmOR24	48	0.45	41.18
*OfurOR13*	k38_268795 k42_1382487	113	AB186515.1|BmorOR13	92	2.32	46.82
*OfurOR14*	k72_633491	390	AB472096.1 |BmOR14	30	0.16	20.58
*OfurOR15*	k46_799430	313	DAA05974.1|BmOR15	40	0.65	3.77
*OfurOR16*	k58_927836	387	AB472097.1|BmOR16	64	0.31	2.87
*OfurOR17*	k74_262490	315	XM_004927118.1|BmOR-1 like	28	0.02	0.42
*OfurOR18*	k42_287214	>127	AB472099.1 | BmOR18	65	0.06	0.35
*OfurOR19*	k68_709537	252	AB472131.1 | BmOR54	58	0.61	3.7
*OfurOR20*	k38_1276784 k44_1248125	243	AB472100.1| BmOR20	46	0.31	3.51
*OfurOR21*	k38_1420394	109	BK005929.1 |BmOR21	55	NPM	NA
*OfurOR22*	k38_1042411	381	AB472102.1|BmOR22	63	0.27	NA
*OfurOR23*	k56_857693	347	AB234358.1|BmOR candidate	32	0.7	NA
*OfurOR24*	k52_33406	133	AB472104.1 | BmOR24	64	0.38	NA
*OfurOR25*	k64_307418 k72_53460	333	AB472105.1 | BmOR25	39	0.48	NA
*OfurOR26*	k40_1068762	88	AB472106.1 | BmOR26	45	0.09	NA
*OfurOR27*	k64_783825	333	AB472107.1|BmOR27	72	0.32	NA
	k68_581233					
*OfurOR28*	k62_822037	>202	BAH66346.1|BmOR51	56	0.29	NA
*OfurOR29*	k74_614504 k58_933385	352	AB472109.1|BmOR29	64	0.78	NA
*OfurOR30*	k44_1297835	402	AB472137.1| BmOR60	69	0.6	NA
*OfurOR31*	k64_794506	245	XM_004927118.1| BmOR1-like	28	0.14	NA
*OfurOR32*	k50_1104273	202	AB472111.1|BmOR32	51	0.45	NA
*OfurOR33*	k46_1238743	421	EHJ75140.1BmOR65	28	0.63	NA
*OfurOR34*	k48_1039646	181	AB472096.1 |BmOR14	54	0.42	NA
*OfurOR35*	k48_498248	96	BK005941.1 | BmOR35	65	0.82	NA
*OfurOR36*	k58_911449	213	AB472115.1|BmOR36	62	0.93	NA
*OfurOR37*	k56_960372	316	AB472141.1 |BmOR64	49	0.56	NA
*OfurOR38*	k38_1364355	306	AB472143.1| BmOR66	55	0.42	NA
*OfurOR39*	k80_497904 k70_664132 k70_679125	384	BK005941.1 | BmOR35	56	-	NA
	k80_496091					
*OfurOR40*	k38_1514766	224	XM_004933061.1| BmOR85b-like	63	0.56	NA
*OfurOR41*	k78_551680	203	AB472136.1 |BmOR59	66	0.55	NA
*OfurOR42*	k56_473159	225	AB234353.1 | BmOR42	60	0.35	NA
*OfurOR43*	k60_881584	296	BK005941.1 |BmOR35	61	0.64	NA
*OfurOR44*	k38_1299672	289	AB472121.1 | BmOR44	80	0.47	NA
*OfurOR45*	k48_325096	66	AB234352.1 | Candidate BmOR	72	0.71	NA
*OfurOR46*	k44_853306	105	AB472115.1| BmOR36	54	0.51	NA
*OfurOR47*	k38_981744	209	EHJ78030.1| *Danaus plexippus* OR29	80	0.81	NA
	k38_508994					
*OfurOR48*	k46_790610	111	BK005932.1 | BmOR24	32	0.5	NA
*OfurOR49*	k56_663134	409	EU779802.1|BmOR49	71	0.59	NA
	k48_498248 k70_676694					
*OfurOR50*	k50_850666	154	AB472127.1 1|BmOR50	33	0.1	NA
*OfurOR51*	k64_318559	237	AB472140.1|BmOR63	71	-	NA
*OfurOR52*	k70_329478	208	AB472102.1 |BmOR22	59	0.55	NA
*OfurOR53*	k38_827254	118	XM_004933061.1| BmOR85b-like	44	0.28	NA
*OfurOR54*	k58_760742	142	BK005924.1 | BmOR15	45	0.04	
*OfurOR55*	k66_754859	122	AB472127.1|BmOR50	37	0.16	
*OfurOR56*	k58_431810	281	AB472133.1|BmOR56	68	-	
	k38_1420906					
	k80_494086					

Constituent contigs are provided along with the number of derived amino acid residues encoded in the full or partial CDS. The Top database hit for each *OfurOR* gene corresponding the *Bombyx mori* ortholog are listed along with percent protein identity (% ID), and do not represent ortholog predictions as predicted in Fig 4). Comparative gene expression is reported as a ratio of male to female (M:F) transcript levels estimated by depth among RNA-seq reads (RSq) and real-time qPCR (qRT). NPF = not present in female libraries (exclusive male expression); NPM = not present in male libraries (exclusive female expression). NA = data not available.

### Analysis of differential gene expression

**Fig 1:**
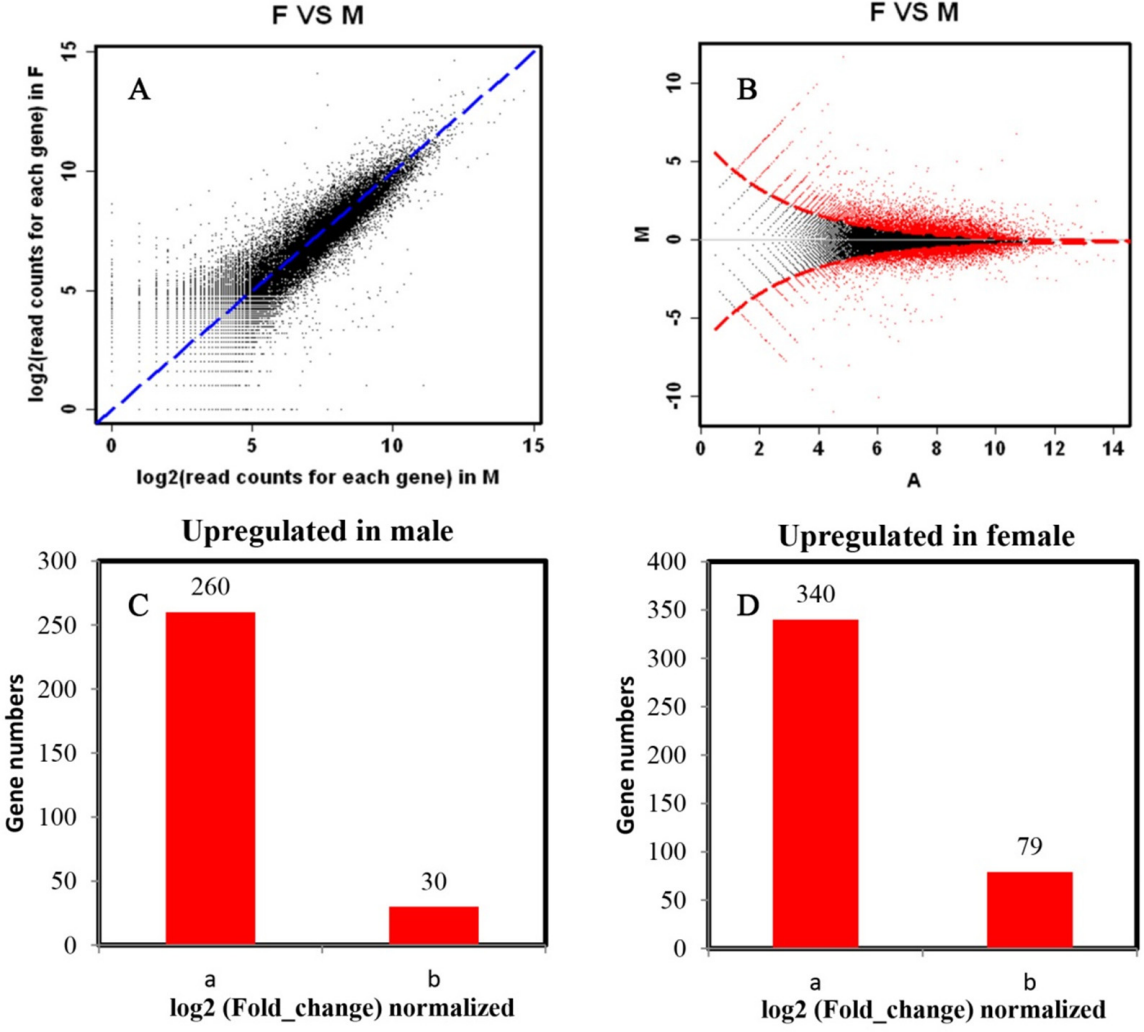
Differentially-expressed genes identified between male and female *Ostrinia furnacalis* antennae. **A**. Scatter plot of pairwise normalized abundance of each transcript among reads obtained from male- and female-specific antennal cDNA libraries. **B.** MA-plot-based method estimates of differential gene expression using a random sampling model, where the red data points represented as red dots indicate genes that show a comparatively significant level of up-regulation in females (above horizontal) and male antennae (below horizontal) (*P* ≤ 0.001). “M” is the binary logarithm of the intensity ratio and “A” is the average log intensity for a dot in the plot. **C.** Genes up-regulated in male antennae; **D.** Genes up-regulated in female antennae. All comparisons of differential gene expression based on log2(fold_change) normalized > 2 or log2(fold_change) normalized > 5.

Analysis of the depth of reads mapped to the reference transcriptome predicted 6,248 significantly DEGs between male and female antennae when a low DESeq cutoff *P*-value < 0.001; 2,122 up-regulated genes (Ratio > 2896 and Ratio > 4305) and 4,126 down-regulated genes (Ratio > 2896 and Ratio > 4305). DESeq results also indicated that male and female antennae respectively showed 260 and 340 up-regulated genes when a higher stringency cutoff of a log_2_(fold-change) ≥ 2 was applied (**Fig 1**). However, adjusting the value of normalized log_2_(fold-change) used as the cutoff was increased to ≥ 5 resulted in the decrease of comparatively up-regulated genes to 30 and 79 respectively in male and female antennae (**Fig 1**C and 1D).

**Fig 2:**
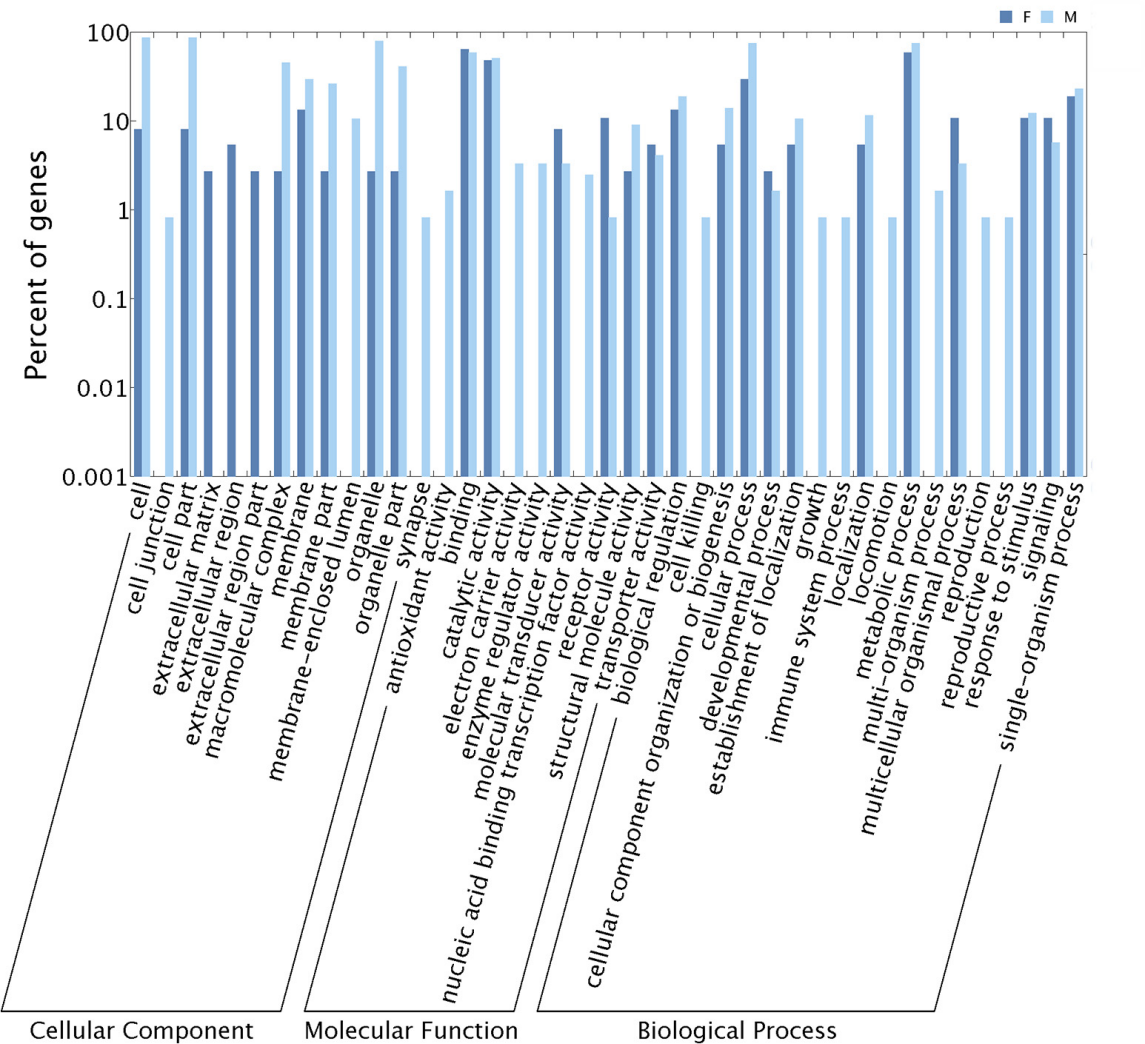
Gene ontology (GO) classifications for the differentially expressed genes in male and female *O*. *furnacalis* antennae.

Corresponding BLASTx annotations obtained for the 30 putatively female-biased transcripts included female-specific genes yolk polypeptide 2 and egg protein 80 (**S4 Table**). Comparatively, male biased antennal transcripts were placed in 11 GO subcategories not annotated for female antennal transcripts (**Fig 2)** [(antioxidant activity (1.65%), electron carrier activity (3.31%), enzyme regulator activity (3.31%), transcription factor activity (2.48%), cell killing (0.83%), growth (0.83%), immune system process (0.83%), locomotion (0.83%), multi-organism process (1.65%), reproduction (0.83%), and reproductive process(0.83%)]. Under high stringency cutoff of log2(fold-change) > 5, 8 OR genes (*OfurOR1*, *OfurOR6*, *OfurOR7*, *OfurOR8*, *OfurOR9*, OfurOR12, *OfurOR15* and *OfurOR20*), as well as 1 OBP (*OfurPBP3*) were significantly up-regulated in male compared to female antennae.

**Fig 3:**
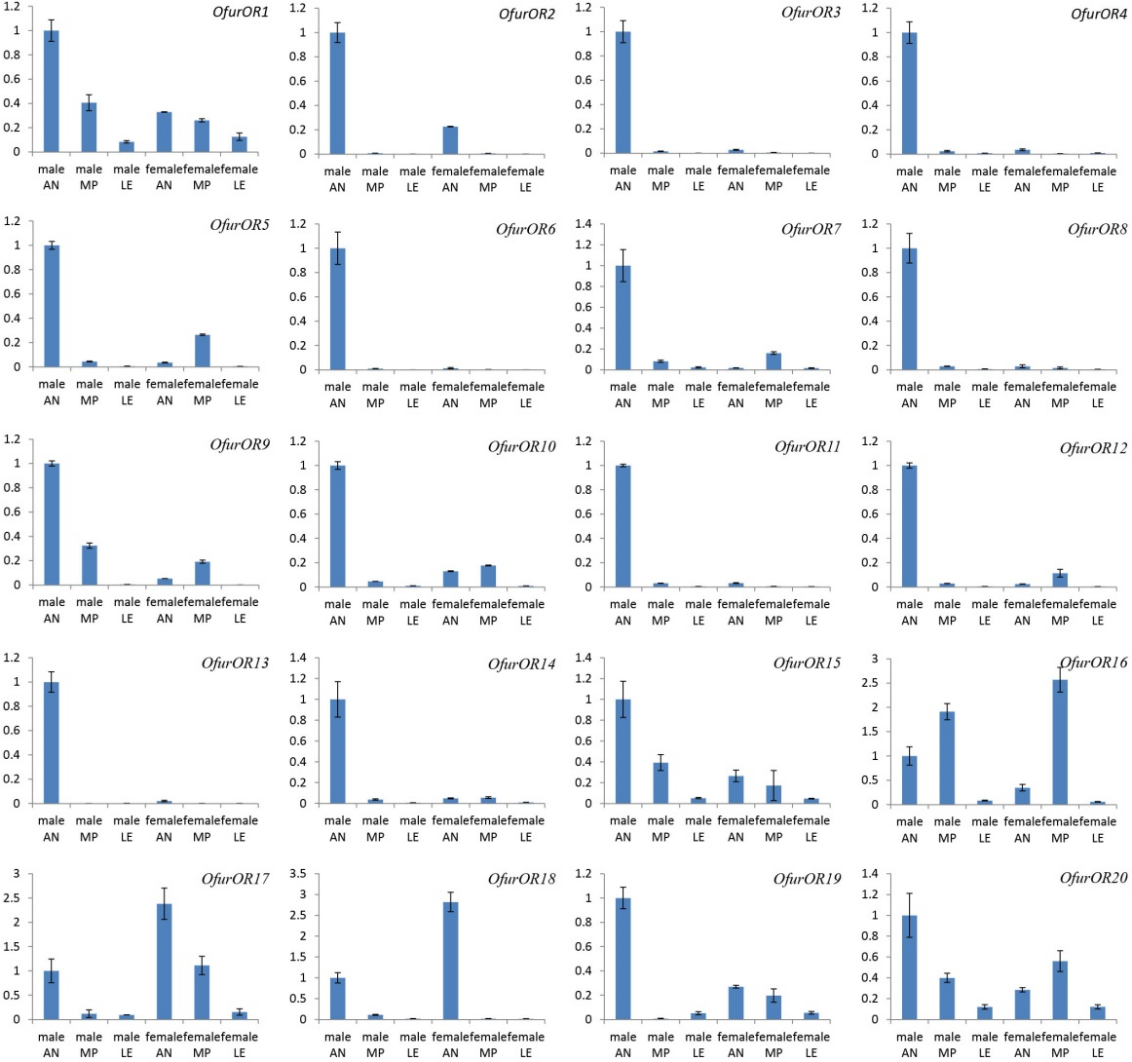
Real-time RT-qPCR estimates of OR transcript levels Assays run using antennae (AN), maxillary palpus (MP) and leg (LE) tissues from in male and female *O*. *furnacalis* estimated from normalized data using the delta delta C_T_ method. Standard error for each sample reported across technical replicates performed in triplicate.

**Fig 4:**
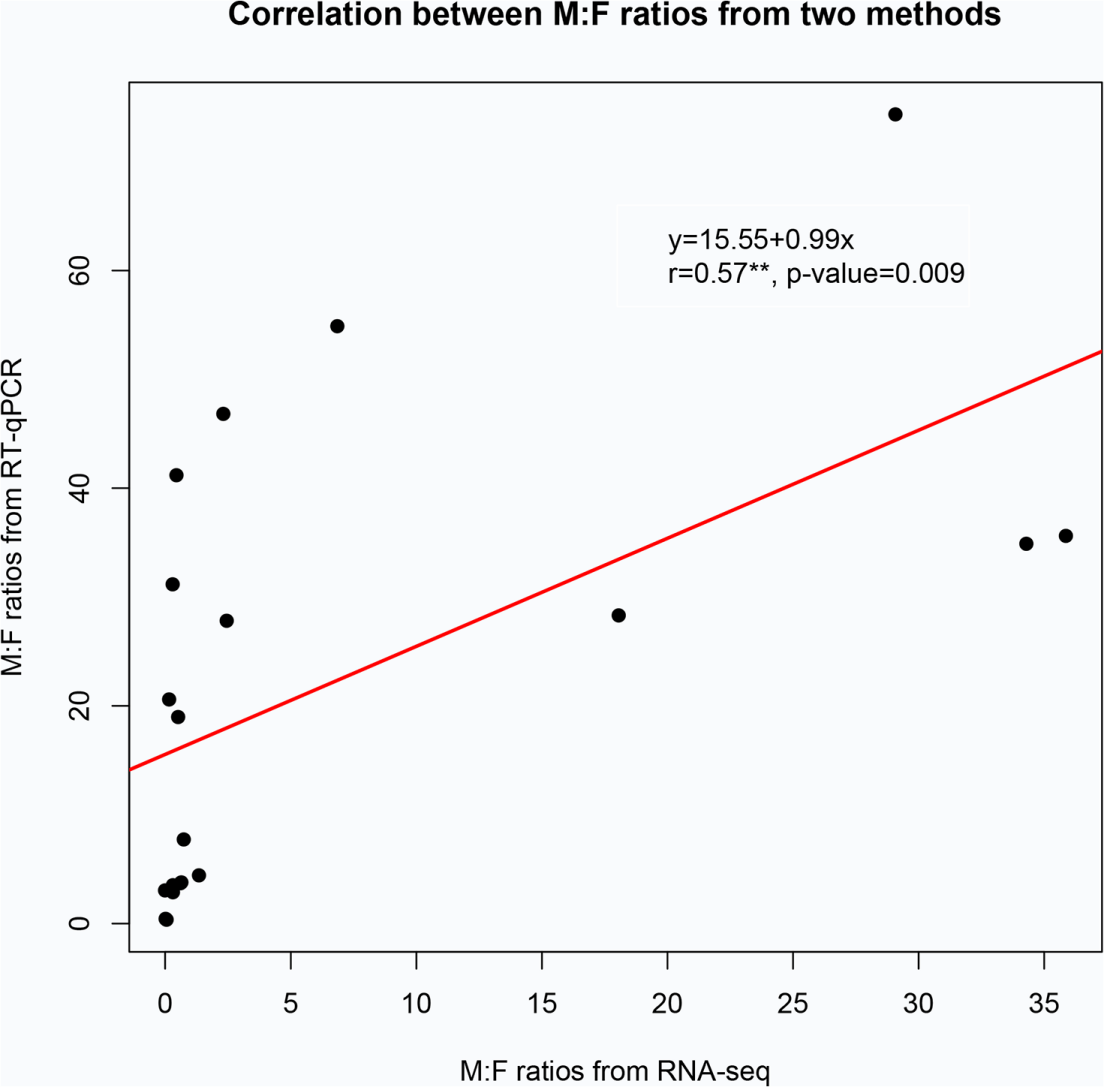
The correlation of estimated *OfurOR1*-*20* transcript levels between RNA-seq and RT-qPCR methods.

Transcript levels were estimated between male and female *O*. *furnacalis* by RT-qPCR for *OfurOR1* to *OfurOR20* using antennae-, maxillary palps- and leg tissue-derived cDNA as template. These results showed that transcripts for *OfurOR2*, *3*, *4*, *6*, *8*, *11*, *13*, *14*, and *18* were only detected in antennal tissues, whereas transcript for all the remaining *Ofur*OR genes were also present in maxillary palpus and leg tissues. Most *OfurOR* genes showed higher estimated expression levels in antennal tissues, with the exception of *OfurOR16*, *20* (**Fig 3**). Transcripts that showed highly biased expression in male antennae included sex pheromone receptor subfamily members, *OfurOR1*, and *3* to *8*, as well as *OfurOR2*, *9* to *15*, *19*, and *20* (**Fig 3**). In contrast, *OfurOR17* and *18* transcript levels were higher in female compared to male antennae (Table 1). Comparisons of relative level of each transcript showed statistically significant differences between male and female antennal tissue (T-test *P*-values ≤ 0.0023), and included an estimated 3-fold greater level of *OfurOR2* in male antennae (*P*-value = 0.0001; remaining results not shown). Comparison of the relative difference in C_T_ values estimated from RT-qPCR experiments to log_2_(fold-change) estimates from RNA-seq data demonstrated that the two methods are not in 100% agreement across 20 *OfurOR* genes that were tested (Table 1). Although inconsistencies were observed, the male-specific sex pheromone receptor class (*OfurOR1*, *3* to *8*) and female biased class (*OfurOR17* and *18*) were highly analogous between the two methods. Correlation between M:F ratios between RNA-seq and RT-qPCR showed an r = 0.57 and *P*-value = 0.009 (**Fig 4**).

### Phylogenetic analyses of olfactory-related proteins

A 102 amino acid long consensus alignment was generated for 71 ORs from *B*. *mori* [40,61,62], *Orco* from *C*. *punctiferalis* and 56 putative *OfurOR*s identified in this study. This alignment showed a (Ser/Ala)-Tyr-(Ser/Thr) C-terminal motif among *OfurOR10*, *13*, *16*, *26*, *27*, *34* and *37*, which is believed to be conserved among *B*. *mori* ORs with female-biased expression [40].

**Fig 5:**
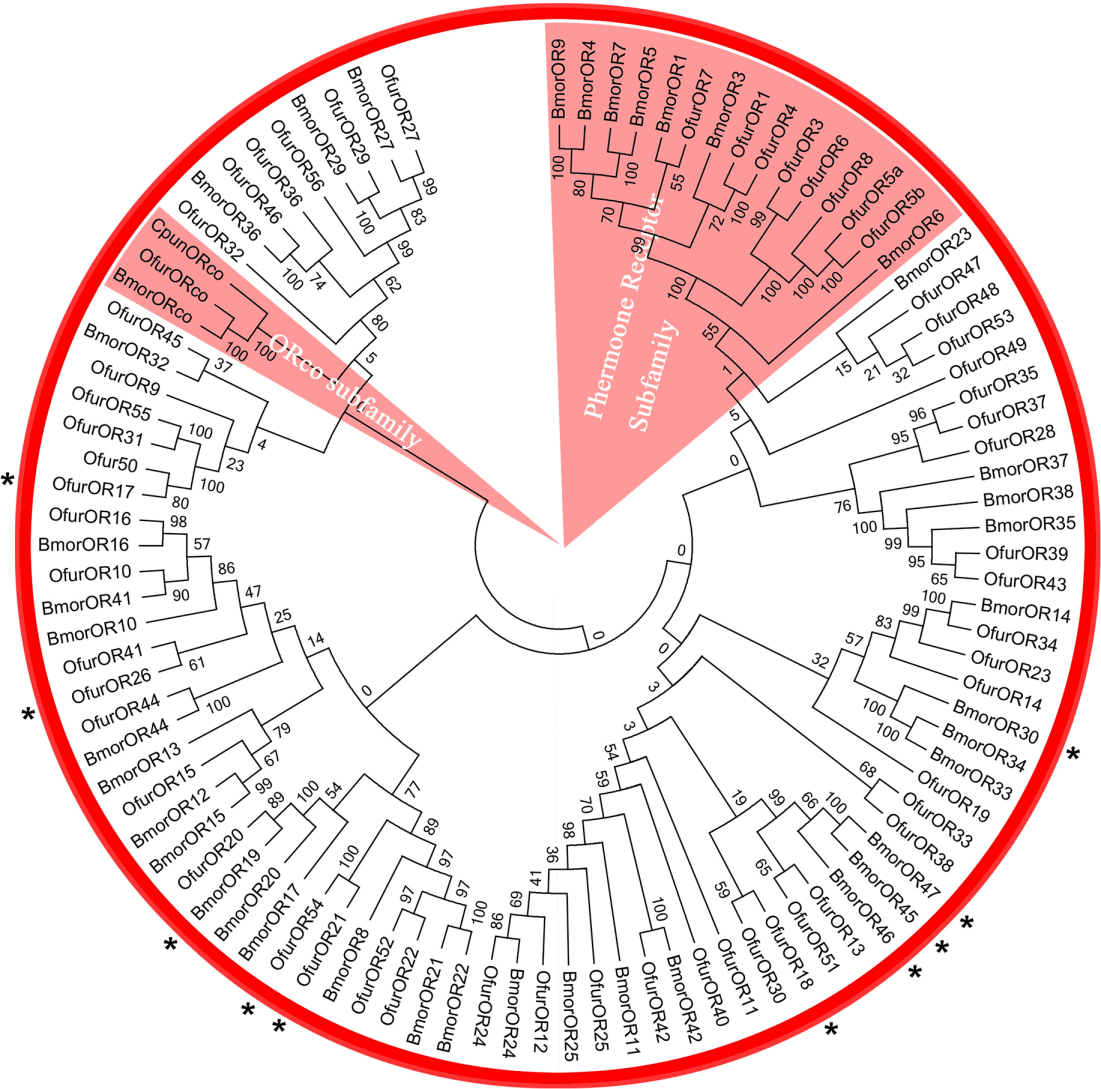
Phylogenetic relationship among odorant receptor (OR) orthologs. Lepidopteran OR gene family relationships were constructed by a Neighbor-Joining (NJ) method. The OR unigenes of *Ostrinia furnacalis* (abbreviated *Ofur*) with are marked with red spot, and Bmor and Cpun are respective abbreviations for *Conogethes punctiferalis* and the model species *Bombyx mori*. Highlighted sectors are labeled for the odorant receptor co-receptor (*ORco*; *OR2*) genes and pheromone receptor subfamily members. Transcripts showing female-biased expression are indicated with an asterisk (*).

Phylogenetic reconstruction based on this alignment indicated that *OfurOR2*/*Orco* clustered with known odorant receptor co-receptors from *B*. *mori* and *C*. *punctiferalis*. *OfurOR1*-*8* proteins clustered into a well-supported monophyletic clade along with sex pheromone receptors from *B*. *mori*. Strong node support could not be obtained in all cases for one-to-one relationships between *O*. *furnacalis* and *B*. *mori* OR orthologs (**Fig 5**), but clustering of orthologs was observed in several instances (e.g. between *Bmor*OR16 and *OfurOR16*, and *BmorOR44* and *OfurOR44*) which might indicate that these ORs are derived from a common ancestral gene. Results also indicated that lineage-specific amplification of ORs may have taken place within *O*. *furnacalis*, where, for example *OfurOR14*, *20* and *23* all appear to share *BmorOR23* as a most-recent common ancestral gene.

**Fig 6:**
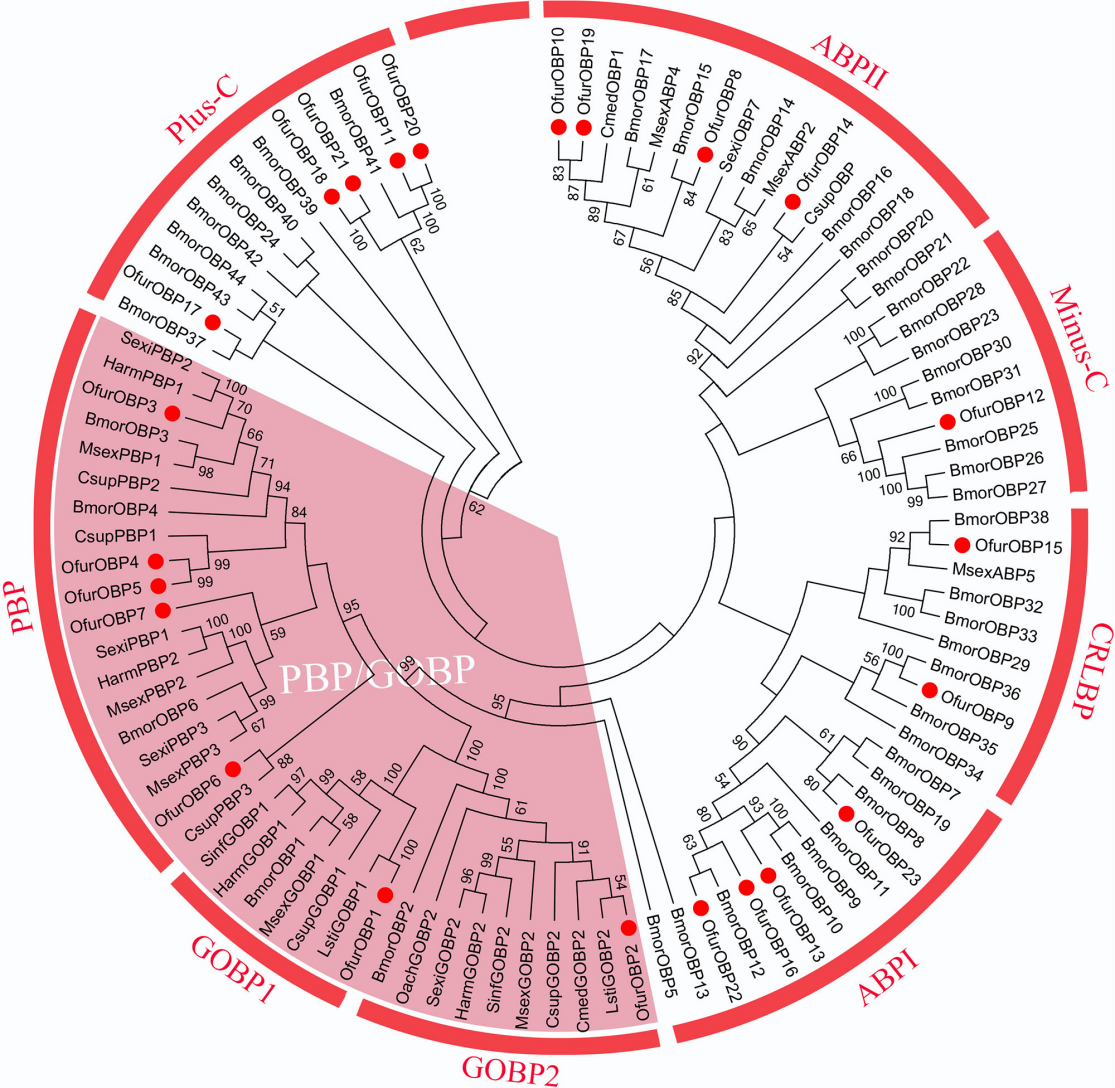
Neighbor-Joining (NJ) estimated phylogenetic relationship of odorant binding protein (OBP) orthologs from species of Lepidoptera. Pheromone binding protein (PBPs)/general odorant binding proteins (GOBPs), chemical sense-related lipophilic ligand-binding proteins (CRLBPs), antennal binding protein group I (ABPI) and II (ABPII), and atypical Plus-C and Minus-C subfamilies are indicated. Abbreviations; Csup for *Conogethes suppressalis* (Lepidoptera: Crambidae), Msex for *Manduca sexta* (Lepidoptera: Sphingidae) Harm for *Helicoverpa armigera* (Lepidoptera: Noctuidae), Sexi for *Spodoptera exigua* (Lepidoptera: Noctuidae), and Bmor for *Bombyx mori* (Lepidoptera: Bombicidae).

A total of 23 putative OBPs were identified in the combined *O*. *furnacalis* antennal transcriptome, including genes encoding 5 PBP- and 2 GOBP-like proteins (Table A in **S3 Table**). Phylogenetic analysis of *O*. *furnacalis* OBPs indicated that derived *O*. *furnacalis* OBPs clustered into ABPI, ABPII, CRLBP, Minus-C, Plus-C, and PBP/GOBP subfamilies as defined previously for homologous gene products from *B*. *mori* [37] (**Fig 6**). Specifically, *Ofur*PBPs and *Ofur*GOBPs clustered with homologous genes from *B*. *mori*, but one-to-one ortholog relationships were not defined for *O*. *furnacalis* within the pheromone binding protein (PBP) clade. A single member of the Minus-C subfamily, and two CRLBP transcript homologs were identified from the combined *O*. *furnacalis* antennal transcriptome, which is fewer than the 8 and 5 orthologs annotated from the *B*. *mori* genome assembly. Other *O*. *furnacalis* OBP classes similarly lacked putative *B*. *mori* orthologs, which might be due to lack of expression in antennal tissues or under treatment conditions used in this study.

A total of 10 CSPs were identified in the *O*. *furnacalis* transcriptome (Table B in **S3 Table**). Phylogenetic reconstruction predicted that nine of these 10 putative *OfurCSP*s clustered with orthologs from other lepidopteran species with high node support (bootstrap values ≥ 87) (**S1 Fig**). In contrast, *OfurCSP*9 failed to cluster with other insect CSPs.

## Discussion

Tissue-wide analysis of differential gene expression using RNA-seq data provides a seemingly un-precedent opportunity to define tissue-, stage-, and treatment-dependent variation. Comparison of sexual variation in gene expression in antennal tissues conducted in this experiment identified 109 significantly DEGs (**Fig 1**), which included the up-regulated pheromone receptor subfamily of ORs in male antennae. Several novel significantly DEGs were also identified such as those encoding putative senescence and seminal fluid proteins, an ABC transporter, and egg yolk proteins (**S4 Table**). This set of DEGs likely represent valuable candidate genes for understanding cellular processes that vary between the sexes, but are beyond the scope of the present research and admittedly suffer from a disconnect between statistical and biological significance. Specifically, it is likely not advisable to assume that all fold-changes between transcripts have biological consequences or are relevant to differential function of the olfactory system in male compared to female *O*. *furnacalis* without further testing. Regardless, steps toward understanding the molecular function of olfactory system components in species of Lepidoptera is important for deciphering how individuals perceive the environment, and the role of chemosensory reception in adult mate attraction and reproduction, oviposition site selection and host plant preference [1], and was focused on in this study.

A full genome sequence as well as gene expression data has been obtained for the first model species for Lepidoptera, *B*. *mori* [64,65]. The OR gene family in *B*. *mori* contains an estimated 71 paralogs [40,61,62] that have a highly variable primary sequence outside of a semi-conserved C-terminal domain [40] and a 7 transmembrane domains (pfam02949; [34]). Sequence discovery and functional analyses have been critical in determining OR function in *B*. *mori*, where these data have facilitated determination that *BmorOR1* stimulation is sufficient to elicit male sexual response [66], that binding of *BmorOR56* by *cis*-jasmone is capable of mediating moth attraction to mulberry host plants leaves [62], and that female-specific expressed *BmorOR19*, *30*, and *45*–*50* proteins can be stimulated by plant volatiles [61]. Species from the genus *Ostrinia*, including *O*. *furnacalis*, have emerged as models for the study of male sex pheromone detection systems [67] for which the function of antennal-expressed ORs during male detection of female emitted pheromones have been partially elucidated [31,33]. Despite these advances in uncovering the molecular function of *Ostrinia* ORs in male perception of female sex pheromones, little is known regarding the extent (number and diversity), expression, or function of many OR gene family members in *Ostrinia*.

Functional diversification among members of a gene family often coincides with changes in temporal and spatial gene regulation [68], and also involve sex-specific patterns of expression in Lepidoptera. Specifically, regulation of *PBP3* is biased for high expression in male compared to female antennae [16], and analogous male-biased expression was also shown for *O*. *nubilalis OR3*, *4*, *5* and *6* [31]. In conjunction with *in vivo* pheromone response data collected from the *Xenipus* oocyte system, gene expression patterns are suggestive of a functional role for *OR3*, *4*, *5* and *6* in male pheromone response [31,33]. This male-biased transcription appears to be retained among the *B*. *mori* orthologs *OR3*, *4*, *5* and *6* [69]. Comparative genomic analyses suggest that male-biased expression and female pheromone receptor function is retained in this lepidopteran OR subfamily despite species divergence that spans over 100 million years. Analogously, female-biased transcription of OR gene family members is predicted among transcripts in both *B*. *mori* [40,61] and *O*. *furnacalis*. In the present study, analysis of read depth among aligned RNA-seq reads suggested 16.7-, 50.0- and 3.2-fold greater transcript levels respectively for *OfurOR18*, *17*, and *16* in *O*. *furnacalis* female compared to male antennae (Table 1), and patterns were validated by RT-qPCR (**Fig 3**). Significant correlation between RNA-seq and RT-qPCR estimates of transcript levels were observed, although this should be interpreted with caution given the variance between paired estimates (**Fig 4**). Also, *OfurOR54* and *26* comparatively showed 25- and 11-fold up-regulation in female *O*. *furnacalis* antennae that was significant at our highest stringent cutoff (Log_2_(fold-change) > 5.0). Phylogenetic analysis of *B*. *mori* and *O*. *furnacalis* OR orthologs indicate potential orthology between female biased *BmorOR19* and female biased *OfurOR21* and *54*, as well as the clade comprised of female biased *BmorOR45*, *46* and *47* with *OfurOR18* (**Fig 5**). In contrast, the female biased transcripts *OfurOR17* and *26* were not predicted to show any close orthologous relationship with female biased transcripts from *B*. *mori*.

Given that several *B*. *mori* female-biased ORs are capable of binding host plant volatiles [61,62], it is conceivable that *O*. *furnacalis* orthologs may have retained similar functions, but further studies are required to investigate any potential evolutionary conservation of function. Instances in which clear orthology was not predicted between female biased transcripts from *B*. *mori* and *O*. *furnacalis*, it may be hypothesized that similar expression pattern could have evolved within a different set of genes (e.g. identity by state). Since *Ostrinia* females tend to oviposit on a greater diversity of host plants compared to the more mulberry-specific attraction exhibited by *B*. *mori* females it is conceivable that female *O*. *furnacalis* adaptations may have selected additional ORs (potentially *OfurOR17* and *26*) that allow response to volatiles emitted from a greater range host plants. Thus, selection for ORs that respond to different host plant volatiles may have resulted functional diversification of unrelated genes in *Ostrinia*. Alternatively, divergent selection upon the same ancestral genes for binding to different host plant volatiles may resulted in a high degree of change in the protein sequence such that convergence with unrelated ORs may obscure the phylogenetic and orthologous relationships. The response of these *Ostrinia* ORs to different host plant volatiles remains unknown until appropriate function assays are performed, such that putative function in female host plant recognition prior to ovipostion cannot yet be established.

Courtship involves the emission of low-intensity utrasonic waves from specialized wing scales of male *Ostrinia* which causes conspecific females to become motionless [70] and results in increased frequency of male mating success [70,71]. Hair pencils are located on the 8^th^ sternite of *O*. *nubilalis* males, which when presented during courtship with cognate females, were shown to enhance mating success [72] and later discovered to contain cells that produce a blend of hexadecnyl acetates (male pheromone) [73]. These male pheromones are likely detected by potential female mates prior to copulation when females are observed to rapidly move antennae, suggesting that pheromone perception by females may be important for mate selection [74,75]. The mechanisms of female chemoreception of male pheromones remains unknown, but potentially involves neuronal stimulation by female-specific ORs in a system analogous to that which has been elucidated in male antennae. Functional characterization *OfurOR* stimulation in response to male pheromones using those ORs validated as up-regulated in female antennal might provide insight into female sexual acceptance, and may likely be the focus of future studies.

In addition to the role of ORs in eliciting neuronal signals in response to specific volatiles, CSPs and OBPs also may form important components of the chemosensory system way of shuttling hydrophobic volatiles from the peripheral environment to the ORs. PBPs are a type of OBP which are proposed to bind and chaperone sex pheromones across the sensillar lymph [34] and may, although contentious and still unresolved [34], have co-evolved with the OR gene family to provide additional selectivity in the chemosensory system [16]. Our RNA-seq data agree with prior RT-qPCR results that showed *O*. *furnacalis* PBP3 is significantly up-regulated in male antennae (Log_2_(fold-change) = 5.31). Since lepidopteran PBPs can bind ligands that are structurally similar female pheromones females and are expressed in non-pheromone sensitive tissues [76,77], PBPs and OBPs might have a role in chemosensory reception that is independent of sexual response and may have roles in general environmental perceptions.

The Asian corn borer, is widely distributed in countries from China to Australia, including Japan, Korea, and the Philippines, and is highly destructive to cultivated corn plants due to larval feeding damage to leaf, stalk and seed tissue that in-turn causes reduced crop yields. Larval *O*. *furnacalis* are also found on alternative host plants including bell pepper, cotton, hops, millet, pearl millet, foxtail millet, sugarcane, sorghum, and ginger as well as many weedy native plant species [78]. The attraction of female *O*. *furnacalis* to host plants for oviposition in the landscape may be important for understanding host range and for understanding chemoreception in lepidopteran insects. The evolution of female chemoreception that elicits an attraction and oviposition on host plants that are suitable for development of larval progeny is complex (see review [79]), selection of females that oviposit on plant that best support larval development may likely have shaped this female chemosensory system [80,81]. The molecular mechanisms involved in host plant attraction have been shown to potentially involved specific binding by CSPs in hemipteran insects [82] and OBPs in *Drosophila* [8], but the expression of OBPs and CSPs in non-olfactory sensitive tissues shown in this and prior studies might suggest these protein have alternate functions [83]. The capacity of putative *O*. *furnacalis* OBP and CSPs to bind various ligands have yet to be performed, but defining the structure and expression of CSP and OBP, as well and ORs, in this study represents a significant initial step that will allow future investigation of biological function.

## Supporting Information

S1 Fig Phylogenetic tree of candidate of chemosensory proteins (CSPs) from species of Lepidoptera. (DOC)

S1 Table List of oligonucleotide primers used in time quantitative PCR of OR gene transcripts. (DOCX)

S2 Table Functional gene annotations of contigs from the combined antennal reference assembly. (TXT)

S3 Table List of putative OBP (Panel A) and CSP genes (Panel B). (DOCX)

S4 Table Differential gene expression data from RNA-seq experiments. (TXT)

S1 Text Fasta formatted contigs from a combined reference assembly of male and female transcriptome data. (FA)

S2 Text Fasta formatted file of OR gene transcript and derived peptide sequences. (TXT)

## References

[1] Zhou JJ (2010) Odorant-binding proteins in insects In: Gerald L, editor. Vitamins & Hormones. Burlington: Academic Press pp. 241–272. 10.1016/S0083-6729(10)83010-9 20831949

[2] Leal WS, Nikonova L, Peng GH (1999) Disulfide structure of the pheromone binding protein from the silkworm moth, *Bombyx mori* . Febs Letters 464: 85–90. 10611489 10.1016/s0014-5793(99)01683-x

[3] Lartigue A, Campanacci V, Roussel A, Larsson AM, Jones TA, Tegoni M, et al (2002) X-ray structure and ligand binding study of a moth chemosensory protein. Journal of Biological Chemistry 277: 32094–32098. 12068017 10.1074/jbc.M204371200

[4] Vogt RG, Riddiford LM (1981) Pheromone binding and inactivation by moth antennae. Nature 293: 161–163. 18074618 10.1038/293161a0

[5] Kirkness EF, Haas BJ, Sun W, Braig HR, Perotti MA, Clark JM, et al (2010) Genome sequences of the human body louse and its primary endosymbiont provide insights into the permanent parasitic lifestyle. Proceedings of the National Academy of Sciences 107: 12168–12173. 10.1073/pnas.1003379107 20566863 PMC2901460

[6] Hekmat-Scafe DS, Scafe CR, McKinney AJ, Tanouye MA (2002) Genome-wide analysis of the odorant-binding protein gene family in *Drosophila melanogaster* . Genome Research 12: 1357–1369. 12213773 10.1101/gr.239402 PMC186648

[7] Ozaki M, Takahara T, Kawahara Y, Wada-Katsumata A, Seno K, Amakawa T, et al (2003) Perception of noxious compounds by contact chemoreceptors of the blowfly, *Phormia regina*: putative role of an odorant-binding protein. Chemical Senses 28: 349–359. 12771021 10.1093/chemse/28.4.349

[8] Matsuo T, Sugaya S, Yasukawa J, Aigaki T, Fuyama Y (2007) Odorant-binding proteins OBP57d and OBP57e affect taste perception and host-plant preference in *Drosophila sechellia* . PLoS Biology 5: e118 17456006 10.1371/journal.pbio.0050118 PMC1854911

[9] Plettner E, Lazar J, Prestwich EG, Prestwich GD (2000) Discrimination of pheromone enantiomers by two pheromone binding proteins from the gypsy moth *Lymantria dispar* . Biochemistry 39: 8953–8962. 10913308 10.1021/bi000461x

[10] Große-Wilde E, Svatoš A, Krieger J (2006) A pheromone-binding protein mediates the bombykol-induced activation of a pheromone receptor in vitro. Chemical Senses 31: 547–555. 16679489 10.1093/chemse/bjj059

[11] Sato K, Touhara K (2009) Insect olfaction: receptors, signal transduction, and behavior. Results and Problems in Cell Differentiation 47: 121–138. 10.1007/400_2008_10 19083129

[12] Xu W, Xu X, Leal WS, Ames JB (2011) Extrusion of the C-terminal helix in navel orangeworm moth pheromone-binding protein (AtraPBP1) controls pheromone binding. Biochemical and Biophysical Research Communications 404: 335–338. 10.1016/j.bbrc.2010.11.119 21130734 PMC3019287

[13] Damberger FF, Ishida Y, Leal WS, Wüthrich K (2007) Structural basis of ligand binding and release in insect pheromone-binding proteins: NMR structure of *Antheraea polyphemus* PBP1 at pH 4.5. Journal of Molecular Biology 373: 811–819. 17884092 10.1016/j.jmb.2007.07.078

[14] Leal W (2012) Odorant reception in insects: Roles of receptors, binding proteins, and degrading enzymes. Annu Rev Entomol 58: 373–391. 10.1146/annurev-ento-120811-153635 23020622

[15] Große-Wilde E, Svatoš A, Krieger J (2006) A pheromone-binding protein mediates the bombykol-induced activation of a pheromone peceptor *In Vitro* . Chemical Senses 31: 547–555. 16679489 10.1093/chemse/bjj059

[16] Allen JE, Wanner KW (2011) Asian corn borer pheromone binding protein 3, a candidate for evolving specificity to the 12-tetradecenyl acetate sex pheromone. Insect Biochemistry and Molecular Biology 41: 141–149. 10.1016/j.ibmb.2010.10.005 21056664

[17] Xu P, Atkinson R, Jones D, Smith D (2005) Drosophila OBP LUSH is required for activity of pheromone-sensitive neurons. Neuron 45: 193–200. 15664171 10.1016/j.neuron.2004.12.031

[18] Benton R, Vannice K, Vosshall L (2007) An essential role for a CD36-related receptor in pheromone detection in *Drosophila* . Nature 450: 289–293. 17943085 10.1038/nature06328

[19] Carey AF, Carlson JR (2011) Insect olfaction from model systems to disease control. Proceedings of the National Academy of Sciences 108: 12987–12995. 10.1073/pnas.1103472108 21746926 PMC3156210

[20] Wang P, Lyman RF, Mackay TFC, Anholt RRH (2010) Natural variation in odorant recognition among odorant-binding proteins in *Drosophila melanogaster* . Genetics 184: 759–767. 10.1534/genetics.109.113340 20026676 PMC2845343

[21] Arya GH, Weber AL, Wang P, Magwire MM, Negron YLS, Mackay TF, et al (2010) Natural variation, functional pleiotropy and transcriptional contexts of odorant binding protein genes in *Drosophila melanogaster* . Genetics 186: 1475–1485. 10.1534/genetics.110.123166 20870963 PMC2998325

[22] Biessmann H, Andronopoulou E, Biessmann M, Douris V, Dimitratos S, Eliopoulos E, et al (2010) The Anopheles gambiae odorant binding protein 1 (AgamOBP1) mediates indole recognition in the antennae of female mosquitoes. PLoS ONE 5: e9471 10.1371/journal.pone.0009471 20208991 PMC2830424

[23] Nakagawa T, Sakurai T, Nishioka T, Touhara K (2005) Insect sex-pheromone signals mediated by specific combinations of olfactory receptors. Science 307: 1638–1642. 15692016 10.1126/science.1106267

[24] Syed Z, Ishida Y, Taylor K, Kimbrell D, Leal W (2006) Pheromone reception in fruit flies expressing a moth's odorant receptor. Proceedings of the National Academy of Sciences 103: 16538–16543. 17060610 10.1073/pnas.0607874103 PMC1621046

[25] Pelosi P (2005) Diversity of odorant-binding proteins and chemosensory proteins in insects. Chemical Senses 30: i291–i292. 15738163 10.1093/chemse/bjh229

[26] Campanacci V, Lartigue A, Hällberg BM, Jones TA, Giudici-Orticoni M-T, Tegoni M, et al (2003) Moth chemosensory protein exhibits drastic conformational changes and cooperativity on ligand binding. Proceedings of the National Academy of Sciences 100: 5069–5074. 12697900 10.1073/pnas.0836654100 PMC154299

[27] Angeli S, Ceron F, Scaloni A, Monti M, Monteforti G, Petacchi R, et al (1999) Purification, structural characterization, cloning and immunocytochemical localization of chemoreception proteins from *Schistocerca gregaria* . European Journal of Biochemistry 262: 745–754. 10411636 10.1046/j.1432-1327.1999.00438.x

[28] Maleszka R, Stange G (1997) Molecular cloning, by a novel approach, of a cDNA encoding a putative olfactory protein in the labial palps of the moth *Cactoblastis cactorum* . Gene 202: 39–43. 9427543 10.1016/s0378-1119(97)00448-4

[29] Guo W, Wang XH, Ma ZY, Xue L, Han JY, Yu D, et al (2011) CSP and takeout genes modulate the switch between attraction and repulsion during behavioral phase change in the migratory locust. PLoS Genetics 7: e1001291 10.1371/journal.pgen.1001291 21304893 PMC3033386

[30] Takanashi T, Ishikawa Y, Anderson P, Huang Y, Löfstedt C, Tatsuki S, et al (2006) Unusual response characteristics of pheromone-specific olfactory receptor neurons in the Asian corn borer moth, *Ostrinia furnacalis* . Journal of Experimental Biology 209: 4946–4956. 17142683 10.1242/jeb.02587

[31] Wanner KW, Nichols AS, Allen JE, Bunger PL, Garczynski SF, Linn CE, et al (2010) Sex pheromone receptor specificity in the European corn borer moth, *Ostrinia nubilalis* . PLoS ONE 5: e8685 10.1371/journal.pone.0008685 20084285 PMC2801615

[32] Miura N, Nakagawa T, Tatsuki S, Touhara K, Ishikawa Y (2009) A male-specific odorant receptor conserved through the evolution of sex pheromones in *Ostrinia* moth species. International Journal of Biological Sciences 5: 319–330. 19421342 10.7150/ijbs.5.319 PMC2677733

[33] Leary GP, Allen JE, Bunger PL, Luginbill JB, Linn CE, Macallister IE, et al (2012) Single mutation to a sex pheromone receptor provides adaptive specificity between closely related moth species. Proceedings of the National Academy of Sciences 109: 14081–14086. 10.1073/pnas.1204661109 22891317 PMC3435168

[34] Leal WS (2013) Odorant reception in insects: roles of receptors, binding proteins, and degrading enzymes. Annual Review of Entomology 58: 373–391. 10.1146/annurev-ento-120811-153635 23020622

[35] Vogt RG (2003) Biochemical diversity of odor detection: OBPs, ODEs and SNMPs. Insect Pheromone Biochemistry and Molecular Biology: 391–446.

[36] Vieira FG, Rozas J (2011) Comparative genomics of the odorant-binding and chemosensory protein gene families across the Arthropoda: origin and evolutionary history of the chemosensory system. Genome Biology and Evolution. 10.1093/gbe/evr033 PMC3134979 21527792

[37] Gong DP, Zhang HJ, Zhao P, Xia QY, Xiang ZH (2009) The odorant binding protein gene family from the genome of silkworm, *Bombyx mori* . BMC Genomics 10: 332 10.1186/1471-2164-10-332 19624863 PMC2722677

[38] Force A, Lynch M, Pickett FB, Amores A, Yan YL, Postlethwait J, et al (1999) Preservation of duplicate genes by complementary, degenerative mutations. Genetics 151: 1531–1545. 10101175 10.1093/genetics/151.4.1531 PMC1460548

[39] Grosse-Wilde E, Stieber R, Forstner M, Krieger J, Wicher D, Hansson BS, et al (2010) Sex-specific odorant receptors of the tobacco hornworm *Manduca sexta* . Frontiers in Cellular Neuroscience 4: 22 10.3389/fncel.2010.00022 20725598 PMC2922936

[40] Wanner KW, Anderson AR, Trowell SC, Theilmann DA, Robertson HM, Newcomb RD, et al (2007) Female-biased expression of odourant receptor genes in the adult antennae of the silkworm, *Bombyx mori* . Insect Molecular Biology 16: 107–119. 17257213 10.1111/j.1365-2583.2007.00708.x

[41] Grosse-Wilde E, Kuebler LS, Bucks S, Vogel H, Wicher D, Hansson BS, et al (2011) Antennal transcriptome of *Manduca sexta* . Proceedings of the National Academy of Sciences 108: 7449–7454. 10.1073/pnas.1017963108 21498690 PMC3088587

[42] Liu Y, Gu SH, Zhang YJ, Guo YY, Wang GR (2012) Candidate olfaction genes identified within the *Helicoverpa armigera* antennal transcriptome. PLoS ONE 7: e48260 10.1371/journal.pone.0048260 23110222 PMC3482190

[43] Zhang YN, Jin JY, Jin R, Xia YH, Zhou JJ, Deng JY, et al (2013) Differential expression patterns in chemosensory and non-chemosensory tissues of putative chemosensory genes identified by transcriptome analysis of insect pest the purple stem borer *Sesamia inferens* (Walker). PLoS ONE 8: e69715 10.1371/journal.pone.0069715 23894529 PMC3722147

[44] Yin XW, Iovinella I, Marangoni R, Cattonaro F, Flamini G, Sagona S, et al. (2013) Odorant-binding proteins and olfactory coding in the solitary bee *Osmia cornuta*. Cellular and Molecular Life Sciences: 1–11. 10.1007/s00018-013-1308-2 PMC11113457 23512006

[45] Poivet E, Gallot A, Montagné N, Glaser N, Legeai F, Jacquin-Joly E, et al (2013) A comparison of the olfactory gene repertoires of adults and larvae in the noctuid moth *Spodoptera littoralis* . PLoS ONE 8: e60263 10.1371/journal.pone.0060263 23565215 PMC3614943

[46] Simpson JT, Wong K, Jackman SD, Schein JE, Jones SJM, Birol İ, et al (2009) ABySS: A parallel assembler for short read sequence data. Genome Research 19: 1117–1123. 10.1101/gr.089532.108 19251739 PMC2694472

[47] Robertson G, Schein J, Chiu R, Corbett R, Field M, Jackman SD, et al (2010) De novo assembly and analysis of RNA-seq data. Nature Methods 7: 909–912. 10.1038/nmeth.1517 20935650

[48] Fu LM, Niu BF, Zhu ZW, Wu ST, Li WZ (2012) CD-HIT: accelerated for clustering the next-generation sequencing data. BioInformatics 28: 3150–3152. 10.1093/bioinformatics/bts565 23060610 PMC3516142

[49] Rice P, Longden I, Bleasby A (2000) EMBOSS: The European molecular biology open software suite. Trends in Genetics 16: 276–277. 10827456 10.1016/s0168-9525(00)02024-2

[50] Conesa A, Götz S, García-Gómez JM, Terol J, Talón M, Robles M, et al (2005) Blast2GO: a universal tool for annotation, visualization and analysis in functional genomics research. BioInformatics 21: 3674–3676. 16081474 10.1093/bioinformatics/bti610

[51] Zhao W, Liu W, Tian D, Tang B, Wang Y, Yu C, et al (2011) wapRNA: a web-based application for the processing of RNA sequences. BioInformatics 27: 3076–3077. 10.1093/bioinformatics/btr504 21896507

[52] Huang X, Madan A (1999) CAP3: A DNA Sequence Assembly Program. Genome Research 9: 868–877. 10508846 10.1101/gr.9.9.868 PMC310812

[53] Li H, Durbin R (2009) Fast and accurate short read alignment with Burrows-Wheeler transform. BioInformatics 25: 1754–1760. 10.1093/bioinformatics/btp324 19451168 PMC2705234

[54] Mortazavi A, Williams BA, McCue K, Schaeffer L, Wold B (2008) Mapping and quantifying mammalian transcriptomes by RNA-Seq. Nature Methods 5: 621–628. 10.1038/nmeth.1226 18516045 PMC13303166

[55] Audic S, Claverie J-M (1997) The significance of digital gene expression profiles. Genome Research 7: 986–995. 9331369 10.1101/gr.7.10.986

[56] Yang YH, Dudoit S, Luu P, Lin DM, Peng V, Ngai J, et al (2002) Normalization for cDNA microarray data: a robust composite method addressing single and multiple slide systematic variation. Nucleic Acids Research 30: e15 11842121 10.1093/nar/30.4.e15 PMC100354

[57] Wang L, Feng Z, Wang X, Wang X, Zhang X (2010) DEGseq: an R package for identifying differentially expressed genes from RNA-seq data. BioInformatics 26: 136–138. 10.1093/bioinformatics/btp612 19855105

[58] Livak KJ, Schmittgen TD (2001) Analysis of relative gene expression data using real-time quantitative PCR and the 2-[delta][delta]CT method. Methods 25: 402–408. 11846609 10.1006/meth.2001.1262

[59] Pabinger S, Rödiger S, Kriegner A, Vierlinger K, Weinhäusel A (2014) A survey of tools for the analysis of quantitative PCR (qPCR) data. Biomolecular Detection and Quantification 1: 23–33. 27920994 10.1016/j.bdq.2014.08.002 PMC5129434

[60] Development Core Team (2014) R: A language and environment for statistical computing. R Foundation for Statistical Computing, Vienna, Austria. ISBN 3-900051-07-0.

[61] Anderson AR, Wanner KW, Trowell SC, Warr CG, Jaquin-Joly E, Zagatti P, et al (2009) Molecular basis of female-specific odorant responses in *Bombyx mori* . Insect Biochemistry and Molecular Biology 39: 189–197. 10.1016/j.ibmb.2008.11.002 19100833

[62] Tanaka K, Uda Y, Ono Y, Nakagawa T, Suwa M, Yamaoka R, et al (2009) Highly selective tuning of a silkworm olfactory receptor to a key mulberry leaf volatile. Current Biology 19: 881–890. 10.1016/j.cub.2009.04.035 19427209

[63] Tamura K, Peterson D, Peterson N, Stecher G, Nei M, Kumar S, et al (2011) MEGA5: molecular evolutionary genetics analysis using maximum likelihood, evolutionary distance, and maximum parsimony methods. Molecular Biology and Evolution 28: 2731–2739. 10.1093/molbev/msr121 21546353 PMC3203626

[64] Xia Q, Cheng D, Duan J, Wang G, Cheng T, Kumar S, et al (2007) Microarray-based gene expression profiles in multiple tissues of the domesticated silkworm, *Bombyx mori* . Genome Biology 8: R162 17683582 10.1186/gb-2007-8-8-r162 PMC2374993

[65] Consortium TISG (2008) The genome of a lepidopteran model insect, the silkworm *Bombyx mori* . Insect Biochemistry and Molecular Biology 38: 1036–1045. 10.1016/j.ibmb.2008.11.004 19121390

[66] Jiggins CD, Sakurai T, Mitsuno H, Haupt SS, Uchino K, Yokohari F, et al (2011) A single sex pheromone receptor determines chemical response specificity of sexual behavior in the silkmoth *Bombyx mori* . PLoS Genetics 7: e1002115 10.1371/journal.pgen.1002115 21738481 PMC3128102

[67] Lassance J-M (2010) Journey in the *Ostrinia* world: from pest to model in chemical ecology. Journal of Chemical Ecology 36: 1155–1169. 10.1007/s10886-010-9856-5 20835755

[68] Ohno S (1970) Evolution by gene duplication New York, Heidelberg: Springer Verlag. 59–87 p.

[69] Wanner KW, Nichols AS, Walden KKO, Brockmann A, Luetje CW, Robertson HM, et al (2007) A honey bee odorant receptor for the queen substance 9-oxo-2-decenoic acid. Proceedings of the National Academy of Sciences 104: 14383–14388. 17761794 10.1073/pnas.0705459104 PMC1964862

[70] Nakano R, Skals N, Takanashi T, Surlykke A, Koike T, Yoshida K, et al (2008) Moths produce extremely quiet ultrasonic courtship songs by rubbing specialized scales. Proceedings of the National Academy of Sciences 105: 11812–11817. 10.1073/pnas.0804056105 18695227 PMC2575327

[71] Takanashi T, Nakano R, Surlykke A, Tatsuta H, Tabata J, Ishikawa Y, et al (2010) Variation in courtship ultrasounds of three *Ostrinia* moths with different sex pheromones. PLoS ONE 5: e13144 10.1371/journal.pone.0013144 20957230 PMC2949388

[72] Royer L, McNeil JN (1992) Evidence of a male sex pheromone in the European corn borer, *Ostrinia nubilalis* (Hübner) (Lepidoptera: Pyralidae). The Canadian Entomologist 124: 113–116.

[73] Lassance J-M, Lofstedt C (2009) Concerted evolution of male and female display traits in the European corn borer, *Ostrinia nubilalis* . BMC Biology 7: 10 10.1186/1741-7007-7-10 19257880 PMC2671483

[74] Schlaepfer M, McNeil JN (2000) Are virgin male lepidopterans more successful in mate acquisition than previously mated individuals? A study of the European corn borer, *Ostrinia nubilalis* (Lepidoptera: Pyralidae). Canadian Journal of Zoology 78: 2045–2050.

[75] Thanda Win A, Kojima W, Ishikawa Y (2013) Age-related male reproductive investment in courtship display and nuptial gifts in a moth, *Ostrinia scapulalis* . Ethology 119: 325–334.

[76] Krieger J, Raming K, Breer H (1991) Cloning of genomic and complementary DNA encoding insect pheromone binding proteins: evidence for microdiversity. Biochimica et Biophysica Acta (BBA)—Gene Structure and Expression 1088: 277–284. 2001401 10.1016/0167-4781(91)90064-s

[77] Vogt RG, Rogers ME, Franco M-d, Sun M (2002) A comparative study of odorant binding protein genes: differential expression of the PBP1-GOBP2 gene cluster in *Manduca sexta* (Lepidoptera) and the organization of OBP genes in *Drosophila melanogaster* (Diptera). The Journal of Experimental Biology 205: 719–744. 11914382 10.1242/jeb.205.6.719

[78] Nafus DM, Schreiner IH (1991) Review of the biology and control of the Asian corn borer, *Ostrinia furnacalis* (Lep: Pyralidae). Tropical Pest Management 37: 41–56.

[79] Knolhoff LM, Heckel DG (2014) Behavioral assays for studies of host plant choice and adaptation in herbivorous insects. Annual Review of Entomology 59: 263–278. 10.1146/annurev-ento-011613-161945 24160429

[80] Anderson P, Sadek MM, Larsson M, Hansson BS, Thöming G (2013) Larval host plant experience modulates both mate finding and oviposition choice in a moth. Animal Behaviour 85: 1169–1175.

[81] Caparros Megido R, De Backer L, Ettaïb R, Brostaux Y, Fauconnier ML, Delaplace P, et al (2014) Role of larval host plant experience and solanaceous plant volatile emissions in *Tuta absoluta* (Lepidoptera: Gelechiidae) host finding behavior. Arthropod-Plant Interactions 8: 293–304.

[82] Gu SH, Wang SY, Zhang XY, Ji P, Liu JT, Wang GR, et al (2012) Functional characterizations of chemosensory proteins of the alfalfa plant bug *Adelphocoris lineolatus* indicate their involvement in host recognition. PLoS ONE 7: e42871 10.1371/journal.pone.0042871 22900060 PMC3416781

[83] Pelosi P, Zhou J, Ban L, Calvello M (2006) Soluble proteins in insect chemical communication. Cellular and Molecular Life Sciences 63: 1658–1676. 16786224 10.1007/s00018-005-5607-0 PMC11136032

