# Supplementary material for: Male- and Female-Biased Gene Expression of Olfactory-Related Genes in the Antennae of Asian Corn Borer, Ostrinia furnacalis (Guenée) (Lepidoptera: Crambidae)
Source: PLoS One. 2015 Jun 10;10(6):e0128550. doi: 10.1371/journal.pone.0128550 (PMC4463852; doi:10.1371/journal.pone.0128550)
Supplement: S1 Fig — (DOC) [file pone.0128550.s001.doc]

**Figure S8**. **Phylogenetic tree of candidate of chemosensory proteins (CSPs) from species of Lepidoptera**. Species abbreviations used are Ofur: *O. furnacalis*, Msex: *M. sexta*, Bmor: *B. mori*, Hvir: H. virescens, Mbra: *M. brassicae*, Aips: *A. ipsilon*, Harm: *H. armigera*, Hzea: Hass: *H. assulta*, Pxyl: *Plutella xylostella*.
