# Supplementary material for: Male- and Female-Biased Gene Expression of Olfactory-Related Genes in the Antennae of Asian Corn Borer, Ostrinia furnacalis (Guenée) (Lepidoptera: Crambidae)
Source: PLoS One. 2015 Jun 10;10(6):e0128550. doi: 10.1371/journal.pone.0128550 (PMC4463852; doi:10.1371/journal.pone.0128550)
Supplement: S1 Table — (DOCX) [file pone.0128550.s002.docx]

**Table S1.** **Oligonucleotide primers used in real-time quantitative PCR (RT-qPCR) for *Ostrinia furnacalis* odorant receptor (OfurOR) paralogs.**

| **Gene** | **Forward Primer (5’-3’)** | **Reverse Premer (5’-3’)** | **L** |
| --- | --- | --- | --- |
| **OfurOR1** | GTTCAACGTGATGCCATTCG | CGGGAGGCATCTCCTCATT | 65 |
| **OfurOR2** | TACTCCACGCAGCAGGACTTC | GGCCAGCATCTCTTGCTTCT | 120 |
| **OfurOR3** | TCTGAGGCACAACCTTGAGAAC | GGGCAGATCCCCAATATTCA | 70 |
| **OfurOR4** | TGGACCAACGCTGTGCTTATAC | CCAGCGTACCTTCCCAAAGAT | 105 |
| **OfurOR5** | GTATGGCCGGCGGAAATATT | GCTCCCCGAAGACAATGAGA | 100 |
| **OfurOR6** | ATGCAACAGGAATCGCCATT | AGGCAAGTACCCTTCGAAGACA | 105 |
| **OfurOR7** | GCATGGCCATCCAAATTCAT | TCCCTGCTACTTGGCAAACC | 100 |
| **OfurOR8** | GAAGGAGACCCTTTAGCACTGAAT | GTGACAGCTTCTCTCCGAATACATC | 100 |
| **OfurOR9** | AACTGCATGCTGTCGCAACT | ACCAGGAACTACAGTACGCTGATTG | 100 |
| **OfurOR10** | CCTGTGGCAACTTCGTCTGA | CTTATTACTGCTTGGTGTTGCTGAA | 110 |
| **OfurOR11** | CTGATTATCATGACGAGGACACAAA | AAGAATTGATACGACATGCTGCAA | 110 |
| **OfurOR12** | TCTGCAGGCTTGCTTGAAGA | ACGTTGTCTAATGCCGTCATGT | 110 |
| **OfurOR13** | GGTACATGCTCGCTGGTGATC | TCGGACGCTCATCAAATCAA | 80 |
| **OfurOR14** | TTGTGAACACGTGTATAGCCAGTTG | TCACGGCTTCCCCACAAATA | 120 |
| **OfurOR15** | GCGAAAGCTGAATAAATGTTTGAA | TGTAACATCCCATGAACAACCAA | 185 |
| **OfurOR16** | CGCAAGCTGCAAGGATCAA | AGACACTGGCATCCACATATCAA | 140 |
| **OfurOR17** | TCAGGGCCGTTTCCAATATAA | CCCAGTTGAAAGCATCACTTACG | 125 |
| **OfurOR18** | GTCCTTGGTCATCATCTGCGTAA | GGTAACAGTAGAGGAAGACCTGCAT | 125 |
| **OfurOR19** | TGCCGGTGGTTGTCTATTGTT | CCAACCGCACGAATAAACG | 110 |
| **OfurOR20** | CGGCTCCTTGCTGTTCCAT | TAGCAGCATTTTTGCAGGGTAA | 110 |
