# Supplementary material for: Male- and Female-Biased Gene Expression of Olfactory-Related Genes in the Antennae of Asian Corn Borer, Ostrinia furnacalis (Guenée) (Lepidoptera: Crambidae)
Source: PLoS One. 2015 Jun 10;10(6):e0128550. doi: 10.1371/journal.pone.0128550 (PMC4463852; doi:10.1371/journal.pone.0128550)
Supplement: S3 Table — (DOCX) [file pone.0128550.s004.docx]

**Table A. Candidate *Ostrinia furnacalis* odorant binding protein (OBP) genes**

| **No.** | **GeneID** | **Gene name** | **MacthLength** | **Homology** | **Species** | **E value** | **Male:Female** |
| --- | --- | --- | --- | --- | --- | --- | --- |
|  |  | *Ofur*OBP1/*Ofur*GOBP1 |  |  |  |  |  |
|  | k74_607404 | *Ofur*OBP2/*Ofur*GOBP2 | 489 | gb\|ABY75632.1\|general odorant binding protein 2 | *Loxostege sticticalis* | 1.39E-22 | 0.56 |
|  | k68_704784 | *Ofur*OBP3/*Ofur*PBP1 | 489 | gb\|AAD39443.1\|pheromone binding protein | *Ostrinia nubilalis* | 5.75E-77 | 0.58 |
|  |  | *Ofur*OBP4/*Ofur*PBP2 |  |  |  |  |  |
|  | k80_117568 | *Ofur*OBP5/*Ofur*PBP3 | 501 | gb\|GU828026.1\| pheromone binding protein | *Ostrinia furnacalis* | 5E-08 | 39.64 |
|  | k80_490102 | *Ofur*OBP6/*Ofur*PBP4 | 562 | gb\|ADT78498.1\|pheromone binding protein 4 | *Ostrinia nubilalis* | 5.66E-56 | 0.23 |
|  | k80_500017 | *Ofur*OBP7/*Ofur*PBP5 | 495 | gb\|ADT78504.1\|pheromone binding protein 5 | *Ostrinia furnacalis* | 3.06E-54 | 0.49 |
|  | k76_566805 | *Ofur*OBP8 | 204 | gb\|EHJ65653.1\|odorant-binding protein 1 | *Danaus plexippus* | 7.04E-22 | 0.56 |
|  | k38_705194 | *Ofur*OBP9 | 504 | gb\|EHJ67147.1\|odorant-binding protein 2 | *Danaus plexippus* | 3.10E-75 | - |
|  | k66_747273 | *Ofur*OBP10 | 180 | gb\|AAL60415.1\| antennal binding protein 4 | *Manduca sexta* | 4.42E-25 | 0.68 |
|  | k76_575306 | *Ofur*OBP11 | 333 | gb\|EHJ77172.1\|odorant binding protein | *Danaus plexippus* | 1.30E-20 | - |
|  | k68_72958 | *Ofur*OBP12 | 414 | gb\|AAL60425.1\|antennal binding protein 7 | *Manduca sexta* | 1.83E-36 | 3.49 |
|  | k78_539788 | *Ofur*OBP13 | 414 | gb\|AER27567.1\|odorant binding protein | *Chilo suppressalis* | 5.97E-42 | 0.80 |
|  | k78_110865 | *Ofur*OBP14 | 194 | gb\|ACX53753.1\| odorant binding protein | *Heliothis virescens* | 4.53E-13 | 0.43 |
|  | k80_495064 | *Ofur*OBP15 | 240 | gb\|ACF48467.1\| pheromone binding protein female 1 | *Loxostege sticticalis* | 3.62E-18 | 0..96 |
|  | k66_759133 | *Ofur*OBP16 | 405 | ref\|NP_001153664.1\|odorant binding protein LOC100301496 | *Bombyx mori* | 2.76E-23 | 0.57 |
|  | k80_503000 | *Ofur*OBP17 | 758 | gb\|ADD71058.1\| odorant binding protein | *Chilo suppressalis* | 7.11E-88 | 0.93 |
|  | k72_649343 | *Ofur*OBP18 | 633 | ref\|NP_001157372.1\|odorant binding protein fmxg18C17 | *Bombyx mori* | 1.28E-55 | - |
|  | k64_792905 | *Ofur*OBP19 | 246 | gb\|AAL60415.1\|antennal binding protein 4 | *Manduca sexta* | 2.74E-34 | 0.72 |
|  | k64_794500 | *Ofur*OBP20 | 267 | gb\|EHJ77172.1\| odorant binding protein | *Danaus plexippus* | 3.17E-24 | - |
|  | k64_802437 | *Ofur*OBP21 | 374 | ref\|NP_001157372.1\| odorant binding protein fmxg18C17 | *Bombyx mori* | 1.99E-29 | 0.63 |
|  | k38_406583 | *Ofur*OBP22 | 492 | gb\|ADK47525.1\| odorant binding protein | *Manduca sexta* | 2.68E-25 | - |
|  | k52_1048861 | *Ofur*OBP23 | 387 | gb\|EHJ67764.1\|odorant-binding protein 5 | *Danaus plexippus* | 2.99E-25 | - |

**Table B. Candidate** ***Ostrinia furnacalis* chemosensory protein (CSP) gene**

| **No.** | **GeneID** | **Gene name** | **MacthLength** | **Homology** | **Species** | **E value** | **Male:Female** |
| --- | --- | --- | --- | --- | --- | --- | --- |
|  | k38_1496371 | *Ofur*CSP1 | 156 | dbj\|BAG71921.1\|chemosensory protein 13 | *Papilio xuthus* | 3.21E-19 | - |
|  | k74_605796 | *Ofur*CSP2 | 210 | gb\|ACX47896.1\|chemosensory protein 2 | *Amyelois transitella* | 1.01E-22 | - |
|  | k58_918293 | *Ofur*CSP3 | 381 | gb\|ACX53719.1\|chemosensory protein | *Heliothis virescens* | 1.73E-51 | - |
|  | k58_927017 | *Ofur*CSP4 | 261 | gb\|EHJ67380.1\|chemosensory protein | *Danaus plexippus* | 4.99E-41 | 0.64 |
|  | k74_601261 | *Ofur*CSP5 | 318 | gb\|EHJ67380.1\|chemosensory protein | *Danaus plexippus* | 4.45E-28 | - |
|  | k80_500763 | *Ofur*CSP6 | 279 | dbj\|BAG71919.1\|chemosensory protein 11b | *Papilio xuthus* | 1.46E-19 | - |
|  | k54_1003200 | *Ofur*CSP7 | 183 | gb\|ACX53813.1\| chemosensory protein | *Heliothis virescens* | 1.93E-16 | 1.51 |
|  | k80_494504 | *Ofur*CSP8 | 264 | gb\|AAF16721.1\| sensory appendage protein 4 | *Manduca sexta* | 6.01E-37 | 0.54 |
|  | k76_574558 | *Ofur*CSP9 | 111 | dbj\|BAF91714.1\|chemosensory protein | *Papilio xuthus* | 7.23E -12 | 0.47 |
|  | k70_686291 | *Ofur*CSP10 | 333 | gb\|ACX53817.1\| chemosensory protein | *Heliothis virescens* | 5E-40 | 2.23 |
